# Early Minimally Invasive Removal of Intracerebral Hemorrhage (ENRICH): Study protocol for a multi-centered two-arm randomized adaptive trial

**DOI:** 10.3389/fneur.2023.1126958

**Published:** 2023-03-16

**Authors:** Jonathan J. Ratcliff, Alex J. Hall, Edoardo Porto, Benjamin R. Saville, Roger J. Lewis, Jason W. Allen, Michael Frankel, David W. Wright, Daniel L. Barrow, Gustavo Pradilla

**Affiliations:** ^1^Department of Emergency Medicine, Emory University School of Medicine, Atlanta, GA, United States; ^2^Department of Neurology, Emory University School of Medicine, Grady Hospital, Atlanta, GA, United States; ^3^Department of Neurosurgery, Emory University School of Medicine, Atlanta, GA, United States; ^4^Berry Consultants LLC, Austin, TX, United States; ^5^Department of Biostatistics, Vanderbilt University Medical Center, Nashville, TN, United States; ^6^Department of Emergency Medicine, Harbor-UCLA Medical Center, UCLA, Torrance, CA, United States; ^7^Department of Radiology and Imaging Sciences, Emory University School of Medicine, Atlanta, GA, United States

**Keywords:** intracerebral hemorrhage (ICH), minimally invasive trans-sulcal parafascicular surgery, lobar ICH, deep ICH, minimally invasive surgery (MIS)

## Abstract

**Background:**

Intracerebral hemorrhage (ICH) is a potentially devastating condition with elevated early mortality rates, poor functional outcomes, and high costs of care. Standard of care involves intensive supportive therapy to prevent secondary injury. To date, there is no randomized control study demonstrating benefit of early evacuation of supratentorial ICH.

**Methods:**

The Early Minimally Invasive Removal of Intracerebral Hemorrhage (ENRICH) Trial was designed to evaluate the minimally invasive trans-sulcal parafascicular surgery (MIPS) approach, a technique for safe access to deep brain structures and ICH removal using the BrainPath^®^ and Myriad^®^ devices (NICO Corporation, Indianapolis, IN). ENRICH is a multi-centered, two-arm, randomized, adaptive comparative-effectiveness study, where patients are block randomized by ICH location and Glasgow Coma Score (GCS) to early ICH evacuation using MIPS plus standard guideline-based management vs. standard management alone to determine if MIPS results in improved outcomes defined by the utility-weighted modified Rankin score (UWmRS) at 180 days as the primary endpoint. Secondary endpoints include clinical and economic outcomes of MIPS using cost per quality-adjusted life years (QALYs). The inclusion and exclusion criteria aim to capture a broad group of patients with high risk of significant morbidity and mortality to determine optimal treatment strategy.

**Discussion:**

ENRICH will result in improved understanding of the benefit of MIPS for both lobar and deep ICH affecting the basal ganglia. The ongoing study will lead to Level-I evidence to guide clinicians treatment options in the management of acute treatment of ICH.

**Trial registration:**

This study is registered with clinicaltrials.gov (Identifier: NCT02880878).

## Introduction

Intracerebral hemorrhage (ICH) accounts for 10–15% of all strokes and its prevalence continues to increase with an aging population. ICH-related early mortality rates range from 35 to 52% (1). Among survivors, only 10–25% return to functional independence, with an estimated annual cost of care and productivity losses of approximately 12.7 billion US dollars (2–5). Clinical outcomes following an ICH are negatively affected by the mechanical complications of mass effect leading to concomitant tissue infarction and intracranial hypertension. In addition, the hematoma in the brain parenchyma mediates a secondary inflammatory cascade.

An effective therapy for ICH patients has been elusive despite several well-designed clinical trials focused on surgical evacuation (6, 7), hemostasis augmentation (8), and blood-pressure reduction (9–11), among other interventions, all of which have failed to improve functional outcomes.

The role of surgery in the care of supratentorial ICH has been limited to life-saving clot evacuation, decompressive craniectomy, or a combination of these two procedures. Despite preclinical evidence supporting the role of hematoma evacuation on early correction of intracranial hypertension and prevention of secondary injury mechanisms, surgical intervention for supratentorial ICH remains unproven in randomized clinical trials (12).

Technology and technique development in support of minimally invasive surgery (MIS) has been encouraged by the hypothesis that prior randomized surgical trials failed to demonstrate benefit in part due to cortical and white matter tract injury incurred while accessing the clot with traditional techniques and tools. Indeed, surgical clot evacuation remains appealing based on the existing preliminary data, which continue to support the time-dependent pathophysiology of ICH, and that early removal of clot mitigates injury to surrounding tissue (13–15). Recent reports describing the safety of image-guided catheter placement for aspiration followed by infused thrombolytics in the MISTIE II trial have produced promising results supporting the premise of tissue preservation and the benefit of clot reduction (16).

Since the MISTIE approach was first described, multiple technological advances have occurred in diagnostic imaging, intraoperative frameless navigation, and navigable port-based minimally invasive access. The preliminary experiences reported by Labib et al. and Bauer et al. suggest that minimally invasive trans-sulcal parafascicular surgery (MIPS) is safe, prevents rebleeding, and maximizes clot evacuation (17, 18). In these studies, intervention occurred as early as 16 and 6.2 h, respectively, suggesting that MIPS may be safely performed while preserving eloquent white matter tracts and producing excellent clot reduction, without significant risk of hemorrhage recurrence, even when surgery is performed early.

To further evaluate this strategy, a randomized clinical trial was designed to assess the clinical benefit of a standardized MIS approach. The proposed MIPS approach was developed to provide atraumatic access, high-resolution visualization, and intraoperative tools for maximal clot evacuation and definitive hemostasis. In this article, we describe the design and methods for the Early Minimally Invasive Removal of Intracerebral Hemorrhage (ENRICH) trial and provide the rationale for the choice of design parameters.

## Methods and analysis

### Study objective

The primary objective of the ENRICH trial is to determine if early ICH evacuation using MIPS results in improved outcomes for patients with an ICH. The primary hypothesis is that MIPS will result in an improvement in the 180-day utility-weighted modified Rankin Scale (UWmRS) when compared to patients treated with standard guideline-based management. To address the primary objective, we have designed an adaptive clinical trial with a sample size between 150 and 300 subjects, with frequent interim analyses to determine if early stopping rules are met and if patient population enrichment per hemorrhage location should occur. Randomization between MIPS and standard management groups will be equal and patients will be block randomized by ICH location, anterior basal ganglia (ABG) vs. lobar location as well as Glasgow Coma Score (GCS) with a threshold for stratification <9 vs. ≥9. At each interim analysis, the enrollment scheme defined by hemorrhage location can be adapted based on the *a priori* enrichment plan.

Secondary aims of the study will evaluate the economic and clinical benefits, and the safety of MIPS compared to standard management. The clinical benefit of MIPS is thought to be a function of clot removal while minimizing white matter injury. Therefore, we will determine if the percent volume of ICH reduction is associated with improved UWmRS at discharge, 30, and 90 days between the treatment groups. The economic effect of MIPS for ICH will be evaluated by quantifying the cost per quality-adjusted life-year (QALY) gained through MIPS at 30, 90, 120, and 180 days. Safety of MIPS will be assessed: (1) by determining the effect of MIPS on mortality when compared to standard management at 30 days; (2) by assessing post-operative rebleeding associated with clinical deterioration following MIPS. Rebleeding will be defined by a growth in hemorrhage volume between an initial non-contrast head computed tomography scan (NCCT) and a follow-up NCCT obtained within 24 h of the index NCCT. Lastly, we will evaluate the impact of time from onset of symptoms to MIPS on the UWmRS.

### Study design and method

**Figure 1:**
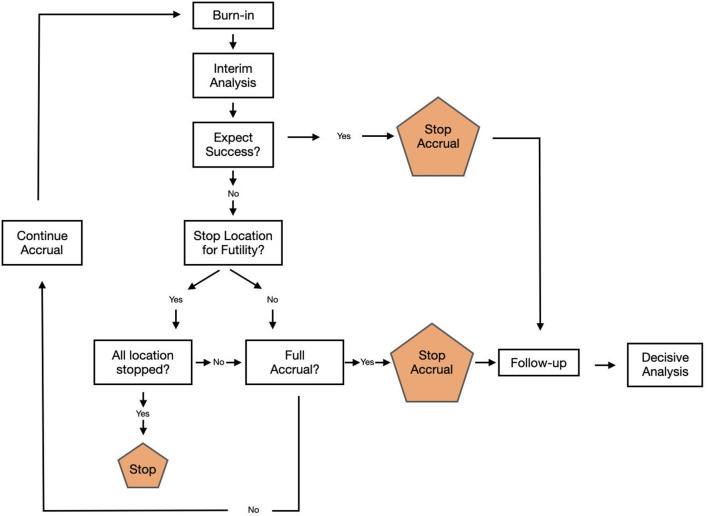
Study flow chart.

ENRICH is an adaptive, two-arm, randomized comparative effectiveness study, where eligible patients are block randomized by ICH location (ABG vs. lobar) and GCS to early ICH evacuation using the MIPS approach plus standard management vs. standard guideline-based management alone. Scheduled interim analyses, beginning after 150 patients have been enrolled, will guide the adaptive sample size and study enrichment (Figure 1). A maximum of 300 subjects will be enrolled.

All randomized subjects are to be treated according to the study protocol, clinical standardization guidelines, and MIPS surgical standardization guidelines. Subjects are followed for 6 months or death (Table 1).

**Table 1:** SPIRIT figure.

	**Study period**								
	**Enrolment**	**Allocation**	**Post-allocation**						**Close-out**
			**Pre-discharge**		**Post-discharge**				
**Timepoint**	*t*_1_	*t*_2_	*t*_3_ = **within 24 h from LKN**	*t*_4_ = **within 7 days from allocation**	*t*_5_ = **discharge**	*t*_6_ = **30 days**	*t*_7_ = **90 days**	*t*_8_ = **120 days**	*t*_9_ = **180 days**
**ENROLMENT**									
Physical assessment, NCCT, CT angiogram, lab, appropriate resuscitation	x								
Eligibility screen	x								
Informed consent	x								
Allocation		x							
**INTERVENTIONS**									
MIPS			x						
Standard treatment			According to CSG						
**ASSESSMENTS**									
Neurologic evaluation, lab, neuroimaging, CSG checklist			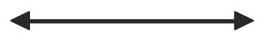						
ICH reduction in association with improved UWmRS					x	x	x		x
Economic effect: QALY						x	x.	x	x
Adverse event monitoring	x		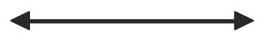		x	x	x	x	x
Safety: mortality			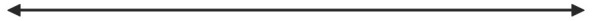						
Safety: rebleeding			x						
mRS	x					x	x		x
HUI						x	x	x	x
NIHSS and GCS	x			x	x				x

### Pre-randomization procedures

**Figure 2:**
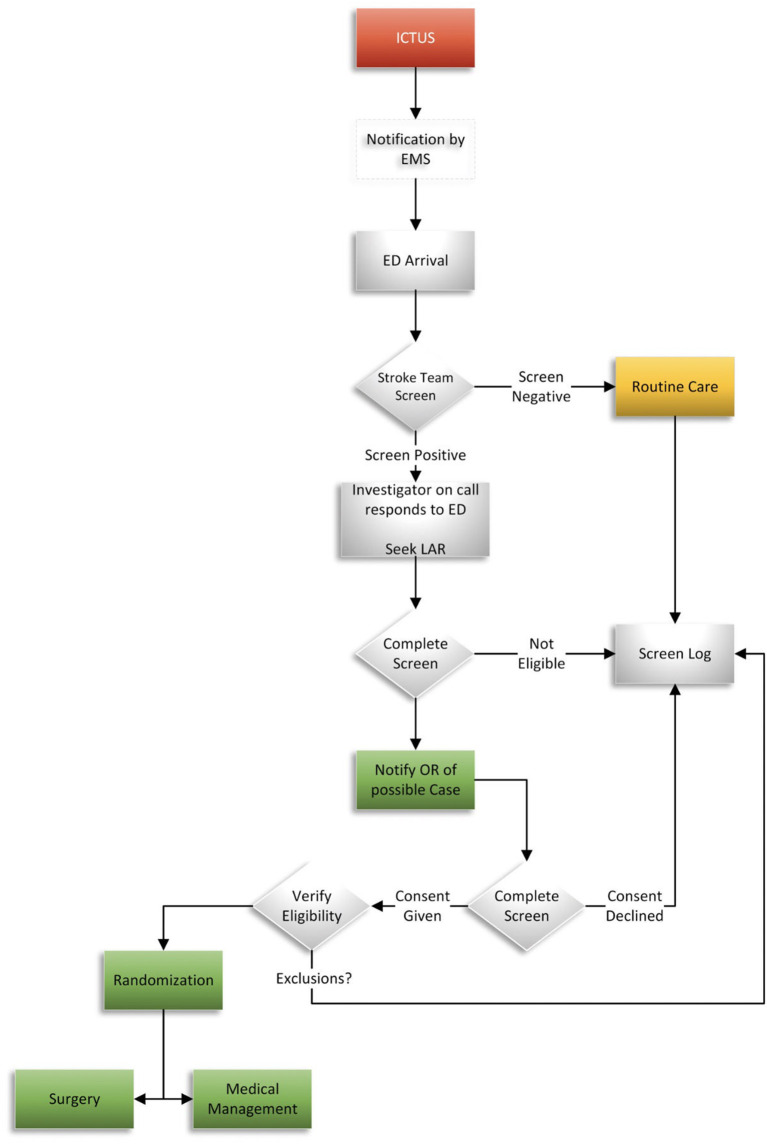
Subject flow chart.

On arrival at the study hospital emergency department (ED), rapid evaluation and treatment will occur per routine including a physical assessment (GCS and National Institutes of Health Stroke Scale-NIHSS), NCCT, head CT angiogram, laboratory evaluation, and appropriate resuscitation (Figure 2). Participating neurosurgeons are instructed to contact the local Investigator on Call (IOC) for subject eligibility.

The IOC will review eligibility criteria and the patient's clinical information (Table 2). Once the patient meets eligibility criteria and informed consent has been obtained from the patient or legally authorized representative, the subject may be randomized using the central electronic data capture (EDC) portal.

**Table 2:** Inclusion and exclusion criteria.

Study inclusion criteria	• Age 18–80 years • Pre-randomization head CT demonstrating an acute, spontaneous, primary ICH • ICH volume between 30 and 80 ml as calculated by the ABC/2 method • Study intervention can reasonably be initiated within 24 h after the onset of stroke symptoms. If the actual time of onset is unclear, then the onset will be considered the time that the subject was last known to be well • Glasgow Coma Score GCS 5–14 • Historical Modified Rankin Score 0 or 1
Study exclusion criteria	• Ruptured aneurysm, arteriovenous malformation (AVM), vascular anomaly, Moyamoya disease, venous sinus thrombosis, mass or tumor, hemorrhagic conversion of an ischemic infarct, recurrence of a recent (<1 year) ICH, as diagnosed with radiographic imaging • NIHSS <5 • Bilateral fixed dilated pupils • Extensor motor posturing • Intraventricular extension of the hemorrhage is visually estimated to involve >50% of either of the lateral ventricles • Primary thalamic ICH • Infratentorial intraparenchymal hemorrhage including midbrain, pontine, or cerebellar • Use of anticoagulants that cannot be rapidly reversed • Evidence of active bleeding involving a retroperitoneal, gastrointestinal, genitourinary, or respiratory tract site • Uncorrected coagulopathy or known clotting disorder • Platelet count <75,000, INR >1.4 after correction • Patients requiring long-term anti-coagulation that needs to be initiated <5 days from index ICH • End stage renal disease • Patients with a mechanical heart valve • End-stage liver disease • History of drug or alcohol use or dependence that, in the opinion of the site investigator, would interfere with adherence to study requirements • Positive urine or serum pregnancy test in female subjects without documented history of surgical sterilization or is post-menopausal • Known life-expectancy of <6 months • No reasonable expectation of recovery, DNR, or comfort measures only prior to randomization • Participation in a concurrent interventional medical investigation or clinical trial • Inability or unwillingness of subject or legal guardian/representative to give written informed consent • Homelessness or inability to meet follow up requirements

### Study treatment

Subjects randomized to MIPS will have the intervention performed as close to the time of randomization as possible. Study protocol requires subjects to enter the OR within 24 h from last known normal (LKN), with a goal of arrival in the OR within 8 h. MIPS is to be performed in accordance with the study surgical manual (Supplementary Datasheet 1). All surgically randomized cases are video recorded, with the first two surgical cases from each site reviewed by the study's lead neurosurgeon (GP) to objectively assess adherence to the surgical protocol. Following surgery, care will follow the Clinical Standardization Guidelines (CSG) for management (Supplementary Datasheet 2).

In the standard treatment arm, subjects will be treated according to the CSG. The decision for surgical intervention with an external ventricular drain for CSF diversion or ICP control/monitoring, decompressive craniectomy, clot evacuation using traditional techniques, or both is left to the treating neurosurgeon. As traditional surgical techniques, such as decompressive hemicraniectomy, remain available to the treating team in the standard management arm, cross-over from this arm to MIPS is strictly not permitted.

### Post-randomization in-hospital assessment

General neurologic evaluation, as assessed by the NIHSS and the GCS, will be monitored daily for the first 7 days following randomization. General laboratory and neuroimaging performed per routine medical care will be collected during this time. A daily checklist will be completed to identify clinical parameters that may be uncorrected and inconsistent with the CSG for care.

### Post-discharge follow up

The outcomes battery includes mRS, NIHSS, GCS, and Health Utilities Index (HUI). The full in-person outcomes battery will occur at 180 days (± 14 days) post-randomization. This assessment will be conducted with both the subject and the primary caregiver/family member to corroborate subject responses. The mRS will also be collected on day 30 (± 7 days) and day 90 (± 14 days) via telephone communication. The 30, 90, and 180-day mRS are audio recorded for blinded adjudication. The 30, 90, 120, and 180-day HUI will be collected by telephone unless visit is conducted in person.

### Primary efficacy analysis

The primary efficacy endpoint is the UWmRS scale at 180 days post-randomization (Table 3). The primary analysis will be performed on all subjects randomized in the ENRICH study. Subjects will be analyzed according to the group they are randomized to, referred to as the intent to treat population. Let Δ be the mean difference in UWmRS between treatment groups (MIPS—standard management) in the ITT population, in which a positive value indicates MIPS benefit.

**Table 3:** mRS values to utility weights mapping.

**mRS**	**Utility weights**
0	1.0
1	0.91
2	0.76
3	0.65
4	0.33
5	0
6	0

The following hypothesis will be tested:

H_0_: Δ ≤ 0, i.e., the mean difference in UWmRS between treatment groups is ≤0
H_1_: Δ> 0, i.e., the mean difference in UWmRS between treatment groups is >0.

If the Bayesian model-based posterior probability of MIPS benefit (i.e., Δ> 0) is ≥0.975, then the study will demonstrate superiority of MIPS vs. standard management.

### Adaptive sample size and enrichment plan

A trial update will be performed when the number of patients enrolled is equal to 150, 175, 200, 225, 250, and 275 patients. The purpose of the trial updates is to determine whether one or two ICH locations meet futility criteria, or whether the current sample size is sufficient to achieve success on the primary outcome. At each trial update, one of the following three decisions will be made:

1) Stop accrual to patients with ICH in either ABG, Lobar, or both locations due to futility.
  a. If accrual stops in only one of the two locations, this creates population enrichment.
  b. If accrual stops in both locations, the trial is stopped for futility.

2) Stop accrual of all patients due to expected success in the pooled ABG/Lobar population. If enrollment is stopped due to expected success, the primary analysis will occur 180 days after the last patient is enrolled.
3) Continue accruing patients without changes.

If the trial does not stop for futility, the primary analysis will occur 180 days after the last patient is enrolled. If enrichment takes place at any point in the trial, and subsequently the primary analysis criteria are met, superiority is only claimed for the location that did not meet the futility criteria, despite the final evaluation including patients from both locations.

In order to stop accrual within a location due to futility, the following two criteria must be met:

1) The Bayesian model-based probability of a clinically meaningful difference (a mean difference of UWmRS of at least 0.075) between surgery and control is <0.20.
2) At least 30 patients have complete 180-day outcomes within the location.

To trigger stopping of accrual for expected success, two criteria must be met:

1) The Bayesian model-based probability of a difference in mean UWmRS utility is ≥0.99.
2) At least 60 patients have completed 180-day outcomes.

### Statistical modeling

The primary analysis will be based on a pre-specified Bayesian hierarchical model which assumes a constant treatment effect across hemorrhage locations, but in which the mean UWmRS in the control group may differ between locations. Missing data will be addressed via Bayesian multiple imputation, in which 90-day UWmRS values are used to inform the likelihood of 180-day values for patients with missing 180-day UWmRS outcomes. The interim updates will incorporate two models: (1) a model equivalent to the primary analysis model for monitoring expected success in the overall study population; and (2) a Bayesian hierarchical model in which the treatment effect is allowed to vary between hemorrhage locations for the purpose of monitoring futility. Both models used in the interim updates will incorporate longitudinal modeling, in which 90-day UWmRS values are used to inform the likelihood of 180-day values for patients with incomplete information.

### Sample size justification

Virtual trial simulations were used to quantify the statistical power and Type I error (Supplementary Datasheet 3). With an adaptive sample size of 150–300 subjects and a base “expected” set of assumptions regarding patient accrual, dropout, distribution of mRS for control and treatment groups, prevalence of ABG/Lobar locations, and treatment benefit in either one or both locations, the Bayesian primary analysis has approximately 90% or greater power for detecting superiority of treatment vs. control for a mean UWmRS difference in both ABG/Lobar locations of 0.15 or larger (i.e., a 15% absolute increase in utility weighted mRS), with an approximate one-sided Type I error <0.025. In addition, the trial design and analysis provide approximately 60–90% power for detecting treatment superiority if there is a mean benefit on UWmRS of 0.15 or larger in one of the two locations.

### Primary safety objective

The primary safety objectives will be evaluated by the rate of mortality and/or rebleeding at 30 days, which will be compared between the two groups using a Pearson's chi-square test and the difference in hemorrhage volume between index CT and 24-h follow-up CT, which will be compared between the two groups using a two-sided *t*-test (or Wilcoxon rank sum test if the normality assumption is violated).

### Secondary endpoints

The secondary endpoints were selected to provide supportive information on safety and efficacy. These secondary endpoints include:

- Postoperative rebleeding associated with deterioration following MIPS (no hypothesis test).
- ICU and in-hospital length of stay (two-sided *t*-test).
- mRS at discharge, 30 and 90 days (ordinal logistic regression; treatment as explanatory variable).
- Impact of percent ICH reduction with MIPS on mRS at 180 days (ordinal logistic regression; %ICH reduction as explanatory variable).
- Impact of end-of-treatment (EOT) volume with MIPS on mRS at 180 days (ordinal logistic regression; EOT volume as explanatory variable).
- Proportion of patients with mRS at 180 days equal to 0, 1, 2, or 3 (chi-square test).
- Ordinal mRS at 180 days (Wilcoxon rank sum test).
- Overall survival through 180 days (log-rank test and Cox proportional hazards model).

### Economic outcome analysis

The ENRICH economic evaluation will assess the primary endpoint: the relative cost-effectiveness of MIPS vs. standard management at 180 days with the effect measured in terms of quality-adjusted life-years (QALYs). QALYs will be calculated from the results of the HUI. Secondary analysis will also: (1) inform how adoption of MIPS will intersect with new and forthcoming Medicare reimbursement policies; and (2) provide evidence on clinical cost-effectiveness.

For the primary endpoint, an incremental analysis will be undertaken to determine the cost per QALY gained through MIPS calculated as: (C_MIP_ – C_std_)/(QALY_MIP_ – QALY_std_) where C_I_ = cost per treatment arm and QALYI = QALYs per treatment arm.

### Safety monitoring

At each Data and Safety Monitoring Board (DSMB) meeting, members will be presented with group comparisons with respect to mortality, occurrence of rebleed and hemorrhage expansion, unexplained and unexpected surgical complications, and other SAEs. The DSMB will also be presented group A–B comparative rates of adverse events to identify any unexpected trends.

An independent medical monitor (MM) will review every SAE for causality and expectedness. For events that are deemed potentially high-risk for future subjects, the DSMB will be provided a report and any necessary additional A–B analysis to make recommendations about study continuation.

The DSMB is a fully independent group of experts selected to advise the ENRICH leadership team, site investigators, and study sponsor and to periodically review and evaluate study data for participant safety, study conduct and progress, and efficacy.

### Rationale for early treatment window

The pathophysiology of ICH is time-dependent; therefore, subjects enrolled in ENRICH and randomized to MIPS will have surgery within 24 h of LKN, with a goal of 8 h. Following the initial ICH, a secondary cascade of inflammation-mediated and pro-apoptotic signaling pathways is initiated, contributing to the poor outcomes (13–15). Previous attempts at ultra-early intervention showed discouraging results using conventional craniotomy (19, 20). Collateral damage of eloquent tissue during surgery remained an important challenge in these studies in addition to poor visualization, and suboptimal hemostasis, all of which we hypothesize have been improved upon with MIPS. Considering the preclinical biological evidence available, a time window of 24 h will allow for early intervention and facilitate institutional and logistic support.

### Rationale for specified eligibility criteria

Eligibility criteria were formulated to investigate a population of ICH patients with high risk for poor outcomes that may also benefit from intervention experiencing a significant event unrelated to a vascular anomaly (21, 22). Patients with a hemorrhage volume <30 ml may benefit from MIPS but any observed relative benefit is likely to be small in magnitude as many of these patients may have reasonable outcomes with supportive medical care. Patients with hematoma volume exceeding 80 ml often have dismal outcomes and poor presenting exams limiting the expected benefit of this intervention. Additionally, clot evacuation for hemorrhages exceeding 80 ml is generally believed to be life-saving and functional improvement is rarely observed (12).

Primary thalamic hemorrhages have been excluded, despite the potential benefit from MIPS, for two principal reasons: (1) thalamic hemorrhages frequently lead to significant midbrain injury, and (2) hemorrhages in this location often have delayed recovery extending beyond 180-days (23).

Direct oral anticoagulants (DOAC) that cannot be reversed are necessarily excluded from the ENRICH trial due to potential untoward risk of hemorrhage expansion and rebleeding. FDA has recently approved agents for reversal of anticoagulation effects of some DOACs (24, 25), as a result, ENRICH excludes patients based on the ability to reverse the effect of the direct inhibitors.

### Rationale for randomization

Randomization minimizes the influence of bias often seen in observational studies of existing therapies. The MIPS technique uses the FDA-cleared BrainPath^®^ and Myriad^®^ (NICO Corporation Indianapolis, IN) devices. Both tools are widely available and have been used in over 40,000 cases of different neurosurgical pathologies including primary and metastatic brain tumors among several others (internal communication, NICO Corporation). Despite this reality, superiority to well-instituted standard management has not yet been established. Therefore, randomization is an ethical and rigorous tool in understanding the potential benefit that MIPS may offer.

### Rationale for the enrichment strategy

Lobar and deep ABG hemorrhages behave differently in prior surgical studies. The STICH trial suggested that more superficial hemorrhages might respond favorably to clot removal in the absence of intraventricular hemorrhage (19). While this hypothesis was not supported in the subsequent STICH II trial, it remains biologically plausible that access to superficial clots may result in less iatrogenic injury (7). As such, we decided to handle these two distinct hemorrhage groups separately so that if we established futility in one group, the investigation into the relative benefits of the other may proceed.

### Rationale for preventing cross over to MIPS

Intentional cross-over from standard management to MIPS is not permitted in this trial. Deteriorating subjects, regardless of randomization assignment may be provided the most aggressive life-saving care available. The most recent national guidelines in the care of the ICH patient detail Class IIb evidence for surgical clot evacuation, decompressive craniectomy, or the combination in the life-saving care of the ICH patient. These procedures, done under traditional techniques may be performed at the discretion of the clinical team, regardless of randomization assignment.

### Rationale for blinded adjudication of outcome

Because this study includes surgical intervention, outcomes cannot be reasonably performed in a blinded manner due to evidence of surgery. Therefore, a unique blinded adjudication process for the mRS was designed to minimize the effect of bias on the primary outcome. The ENRICH team enlisted an experienced Neuropsychologist, who is blinded to treatment allocation, to review audio recordings of subject mRS interviews. Following review of the audio recording the Neuropsychologist enters their assessment into the EDC. Discordance in the mRS value between the site and the Neuropsychologist are adjudicated by case discussion between the blinded and unblinded assessors. The final determination is made by the blinded Neuropsychologist.

### Rationale for cost per quality-adjusted life year gained assessment

ICH is a condition with high morbidity, and often early fatality rate. Should the MIPS approach be superior to standard management it is likely that cost of care of ICH patients may increase in the acute setting, even though the procedure may impact ICU length of stay, ICU-related complications, in-hospital length of stay, or length and complexity of post-acute rehabilitation, all of which may result in overall decreased cost in the long term. By utilizing the QALY metric, the cost can be analyzed in the context of patient outcomes. By convention, an intervention is deemed cost-effective if the treatment is in the range of $100,000–200,000 per QALY gained.

### Rationale for the primary outcome and follow up period

Patient-centered outcomes in stroke research are increasingly desirable. The utility is a measure that expresses the desirability of a specific outcome to a patient. The UWmRS converts the arbitrarily distinguished mRS to a patient-centered scale with distances between the items that better reflect societal and patient beliefs of the desirability of a particular outcome (26). The use of UWmRS also improves statistical efficiency when compared to the ordinal mRS. Statistical analysis of the ordinal mRS often leads to dichotomizing the outcome, reducing the power to detect treatment effects through collapsing important categories of health outcomes, or ordinal analysis, which does not account for the varied importance represented in the distance between each of the categories. The UWmRS may be analyzed as a continuous measure allowing the full use of the obtained data.

A 6-month follow-up term for the primary outcome was selected with the understanding that ICH survivors may continue to improve up to a year or more after the event. ICH trials have moved to a 6-month outcome and we have selected a similar outcome to permit comparison between trials.

### Rationale for site selection

Participating sites have been carefully selected based on prior experience with the MIPS approach, annual ICH volume, partnership between neurocritical care and neurosurgery, and experience of the research team. Selected sites are visited in person by a member of the Scientific Leadership Team (SLT) as well as for Site Initiation Visits prior to full engagement. A minimum of 10 sites will be involved in this study.

### Study organization and funding

Trial design, leadership, and conduct are overseen by the SLT at Emory University and representatives of the sponsor. Berry Consultants supports the SLT with the study's adaptive clinical trial design and one unblinded statistician (BS) performed the interim analyses. The trial sponsor and funder is the NICO Corporation. The sponsor will perform monitoring at all sites to ensure data quality and integrity, and the protection of the rights and safety of subjects through a contract research organization (IQVIA).

## Discussion

### Generalizability

The ENRICH clinical trial has been designed to enhance the care of ICH patients. The overall stroke community is desperately seeking an effective treatment for ICH that directly results in improved functionality. The broad inclusion criteria were designed to focus on those ICH patients with high risk of significant morbidity and mortality. This selection represents a large number of ICH patients and a cohort in whom scientific progress is imperative. Exclusion criteria were also selected with three guiding principles: (1) In whom might early clot evacuation prove beneficial? (2) In whom might the MIPS approach prove harmful? and (3) The ethical imperative to complete the trial with a fullness of scientific understanding that permits the improvement of care of these patients.

The ENRICH trial and the MIPS approach being studied is not being tested for every patient with an ICH, but rather a cohort of patients in whom the risk-benefit profile might lead to overall improvement.

### Expected impact of the proposed trial

There are no Level I evidence-based surgical options in the acute management of ICH. This trial is designed to determine if MIPS for ICH evacuation, performed within 24 h from LKW, results in improved functional outcome and economic benefit. Surgical evacuation following spontaneous ICH is an appealing treatment option; nonetheless, when studied, surgery has not been observed to be efficacious. It is believed that previous negative studies were due in part to trauma that occurs with accessing the ICH, heterogeneity in surgical and medical care, and patient selection. The ENRICH clinical trial improves upon these limitations with technology that permits access to deep structures with minimal trauma. This trial will significantly contribute to scientific literature guiding the optimal management of these medically complex patients.

## Dissemination

The trial results will be disseminated through publications in peer-reviewed journals, presentations at scientific conferences, and media channels.

The datasets generated during the study will be available upon reasonable request.

## Ethics statement

The studies involving human participants were reviewed and approved by Emory University Institutional Ethical Review Boards. The patients/participants provided their written informed consent to participate in this study.

## Author contributions

GP, JR, AH, BS, and RL were responsible for the conceptualization, methodology, and writing the original draft. EP, BS, JA, MF, DW, and DB were responsible for reviewing and editing the protocol. All authors contributed to the article and approved the submitted version.
