# Supplementary material for: Early Minimally Invasive Removal of Intracerebral Hemorrhage (ENRICH): Study protocol for a multi-centered two-arm randomized adaptive trial
Source: Front Neurol. 2023 Mar 16;14:1126958. doi: 10.3389/fneur.2023.1126958 (PMC10061000; doi:10.3389/fneur.2023.1126958)
Supplement: Supplementary file 1 [file Data_Sheet_1.PDF]

Surgical Manual

Following randomization into the surgical arm, a competency trained neurosurgeon will perform the MIPS for clot evacuation with strict adherence to the outlined protocol. Competency training shall include training in a 6-pillar course or equivalent training session, successful participation in the MISPACE registry or the review of 10 cases of MIPS intervention by the surgeon outside of the trial and verification of hospital privileges in stereotactic and image-guided procedures. A slide presentation has been produced describing the procedure and equipment, recommended trajectories, cannulation, resection, hemostasis and decannulation. These materials will be available in the trial website and will be used continuously for training and reference of the surgical teams to maintain standardization of the surgical technique. These materials will be updated as new safety data is acquired. Each site will maintain a log of eligible surgeon(s) along with the date and time of viewing. Each procedure will be recorded and videos will be stored at the site. Initial and selected subsequent videos will be reviewed by the SLT to ensure protocol adherence.

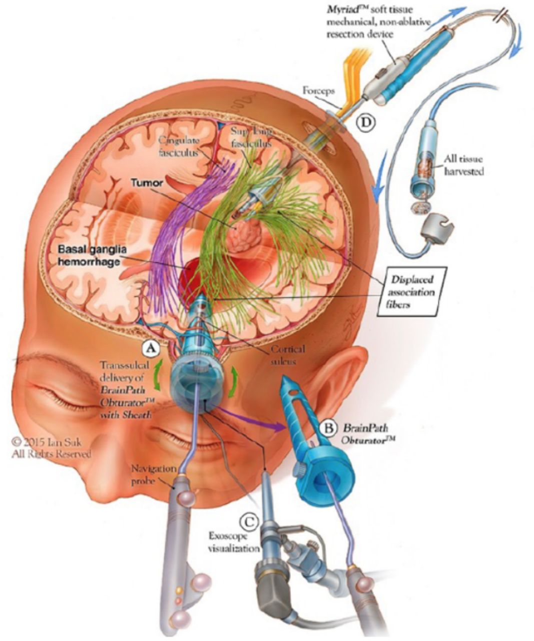

Timing of Surgery – Considering the preclinical biological evidence available, a time window of 24h will allow for early intervention and facilitate institutional and logistic support for intervention. A goal of 8h is preferred and participating sites will be encouraged to improve their time-to-intervention metrics to reach this target.

Image interpretation – Following acquisition of CT angiography DICOM images with a minimum thickness of 1mm will be transferred to the neuronavigational system and three-dimensional images will be reconstructed.

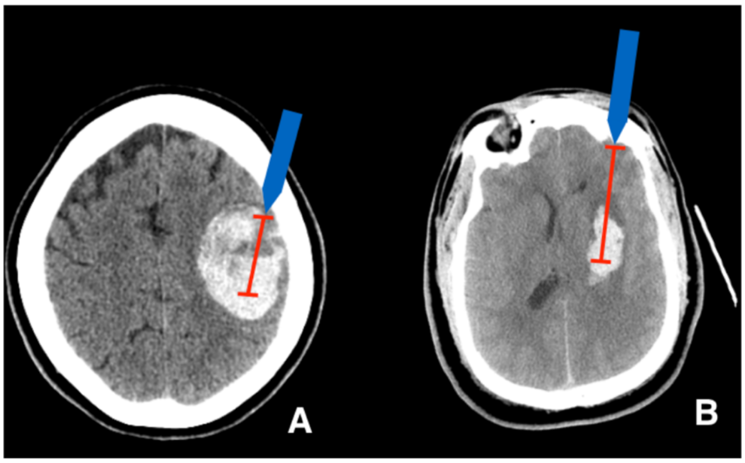

- For anterior basal ganglia (ABG) hemorrhages an anterior frontal sulcus medial to the mid- pupillary line and above the orbital rim will be selected as the entry point (Figure 1). A point approximately 1 cm anterior to the posterior border of the hematoma will be selected as the target for cannulation and a probe's eye view will be evaluated to validate the chosen trajectory (Figure 2A). Adjustments will be made to appropriately cover the largest volume of hematoma and to avoid critical vascular structures. Prior multiple observations of preoperative and postoperative DTI MRI indicate minimal impact of this trajectory on the cingulate fasciculus medially and the superior longitudinal fasciculus laterally.

- For lobar hemorrhages the sulcus closest to the most superficial aspect of the hematoma will be selected and the target will be chosen as described above (Figure 2B). A probe's eye view will be selected to validate the chosen trajectory.

Patient position – Patient position should facilitate an ergonomic cannulation and enable visualization of the navigation and visualization screens. For ABG hemorrhages a supine position with mild head extension is recommended. Forehead crease and eyebrow incisions confer quick access and commonly coincide with the selected entry point in patients with receding hairlines or baldness. For lobar hemorrhages the involved lobe dictates the position.

When possible the long axis of the existing white matter fibers in the anatomical region will be selected as the loading vector for the planned trajectory (Figure 2A).

Anesthetic Plan – Elevated intracranial pressure is advantageous during cannulation to provide adequate resistance to the BrainPath port and deliver the hematoma into the lumen of the port. Mannitol, corticosteroids, hypertonic saline solutions and other maneuvers aimed to decrease ICP are therefore discouraged during cannulation but maybe instituted after clot evacuation has been achieved as deemed necessary by the neurosurgeon. Strict blood pressure control with a systolic blood pressure goal <160mm Hg is recommended. In patients with favorable pre-induction parameters immediate postoperative extubation is desirable.

Navigation Registration & Exoscopic Positioning – Following registration of the stereotactic navigation a flyby should be performed to ensure an unobstructed line of sight to the navigation camera and to estimate the necessary size of the craniotomy to prevent soft-tissue and bony obstructions during cannulation. The Mitaka arm is preferentially positioned on the side of the cannulation and adequate reach of the arm over the planned cannulation area should be ensured. A BrainPath cannula is selected according to the distance to the target, selection should take into consideration additional clearance for the cranial edges, muscle, and skin over which the Shepard's hook is attached.

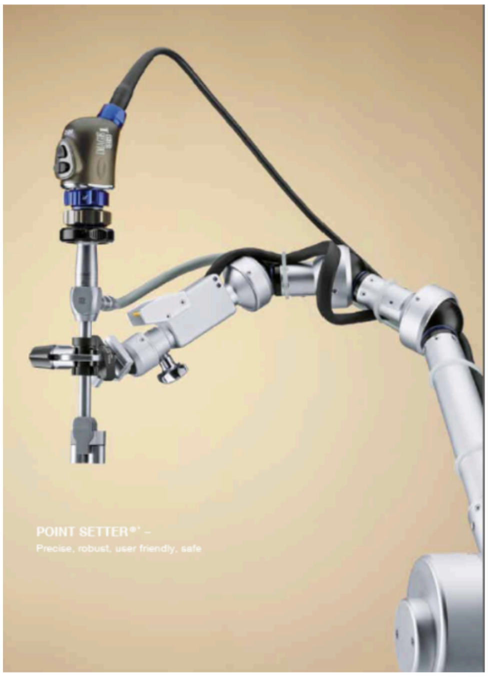

Access – A small incision (usually 4-5cm) is made and a craniotomy that affords adequate range of motion of the BrainPath along the flight plan trajectory is made. A cruciate dural opening is recommended to facilitate containment of the subjacent edematous parenchyma.

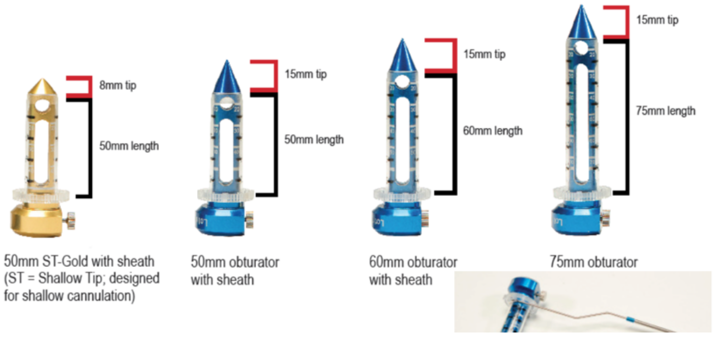

The dural opening should be equal to or slightly less than the diameter of the BrainPath sheath (13.5mm) to promote a tight seal around the port sheath. A small opening (2mm approximately) in the arachnoid is made under exoscopic visualization, every effort is made to preserve surface veins. If a large vascular structure needs displacement during cannulation the arachnoid along this vessel should be opened sufficiently to facilitate mobilization of the vessel without unnecessary tension. The integrated navigation system with obturator and port are then inserted along the preplanned trajectory. The deepest portion of the clot is selected as the target point and cannulated with the BrainPath. Once the obturator is removed the pressure within the sheath is that of atmosphere. A tight seal between the dural edge and the sheath induces a pressure differential that forces the elevated intracranial pressure to deliver the hematoma into the lumen of the port.

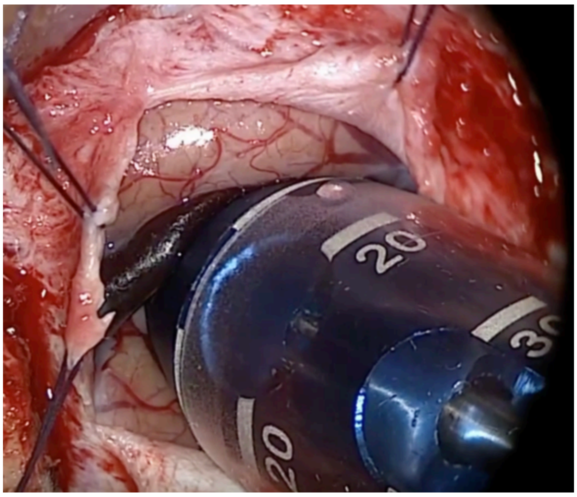

Optics (Exoscopic system) – Adequate visualization at the depth of the port requires an optical system capable of delivering light and magnification along the radial 13.5mm corridor while maintaining sufficient working space for bi-manual dissection. Exoscopic systems deliver light along a divergent pathway generating a cylindrical working corridor and create uniform microscopy by delivering light and magnification along a larger volume of view as opposed to a focal point, which increases tissue differentiation and decreases intraoperative readjustment of the focal length. By remaining at a significant distance from the target the working space inside the port is maximized and standard bimanual microsurgical technique can be practiced.

An exoscopic system with a holding device must be used during clot evacuation to maintain these conditions. Previous experience using microscopy for visualization has been associated with lower rates of evacuation and increased incidence of rebleeding. Enhanced visualization tools (i.e. Storz SPIES Clara & Chroma) that can prevent excessive light absorption from the hematoma and improve accuracy are recommended. Recoding of all procedures performed is required. Video recording and storing capabilities must be available for every procedure.

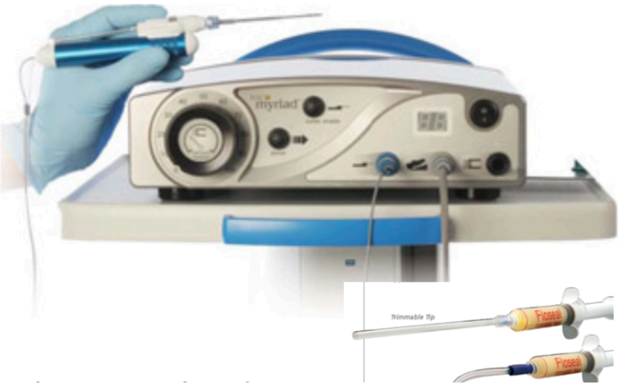

Resection / hemostasis – Clot evacuation proceeds using a combination of standard microsuctions and an automated mechanical resection device. The NICO Myriad device expedites removal of thick fibrous clots and minimizes mechanical trauma to the surrounding tissue. Controlled aspiration with selective cutting prevents blockage of the suction device and decreases operative time. The device should be used for all cases to ensure standardized technique. When visualized, the responsible offending vessel(s) are directly cauterized with bipolar cautery using bimanual technique. Long bayonetted bipolar cautery forceps and standard surgical hemostatic agents such as Surgicel, fibrillar, Gelfoam, FloSeal, or SurgiFlo are recommended. Proper long tip applicators are useful to maintain visibility and increase accuracy. Once the deepest component of the clot is evacuated decannulation proceeds at 1cm intervals with meticulous hemostasis until the sheath is removed.

Adherence Assessment – Protocol adherence will be determined by review of the initial and selected subsequent cases and of the data recorded on the case report forms, which will be

compared with the medical record and other source documentation. Protocol compliance will be reported for each site and for all enrolled patients to the SLT and the DSMB at each scheduled time point. Participating sites with poor compliance will be evaluated by the SLT and a determination will be made regarding further training needs, supporting strategies, or replacement if needed. It is expected that >80% of subjects will have >90% of clot evacuation on postop CT. Percent clot evacuation will be evaluated regularly by the SLT to performance and compliance with protocol.
