# Supplementary material for: Early Minimally Invasive Removal of Intracerebral Hemorrhage (ENRICH): Study protocol for a multi-centered two-arm randomized adaptive trial
Source: Front Neurol. 2023 Mar 16;14:1126958. doi: 10.3389/fneur.2023.1126958 (PMC10061000; doi:10.3389/fneur.2023.1126958)
Supplement: Supplementary file 2 [file Data_Sheet_2.PDF]

Medical Manual

Subjects randomized to either medical management alone or MIPS will be treated according to the Clinical Standardization Guidelines (CSG). The CSG have been adapted by the SLT at Emory University from the 2015 AHA/ASA Guidelines for the Management of Spontaneous Intracerebral Hemorrhage. Whenever clinically feasible the CSG should be followed as they represent a template for care of these subjects. The attending clinician has ultimate responsibility and discretion for treating subjects. The clinician will use his/her best judgment in treating subjects based upon the specific clinical situation and in accordance with GCP.

Blood Pressure Control – Blood pressure will be rapidly controlled to a goal systolic blood pressure (SBP) < 180mmHg within the first 6 hours from arrival. SBP will remain < 180mmHg throughout the course of care. In cases of marked hypertension, SBP > 240 mmHg, an initial 25% reduction is reasonable, prior to targeting < 180 mmHg. Care should be taken in patients with suspected or known ICP elevation to avoid cerebral hypotension by maintaining mean arterial pressure (MAP) > 80mmHg or when ICP monitoring is available, a cerebral perfusion pressure (CPP) > 60mmHg. Systemic hypotension (SBP < 100mmHg; MAP < 60) will be avoided and rapidly corrected in parallel with an investigation into the underlying etiology.

Hemostasis and Coagulopathy – Rapid, targeted, correction of clinically apparent coagulopathy is required.

ICH related to vitamin K antagonist (i.e. warfarin) oral anticoagulants requires withholding the VKA and emergent replacement of vitamin K-dependent coagulation factors. The preferred method of factor replacement is through administration of 4-factor Prothrombin Complex Concentrate (PCC – dose per package insert); if this treatment is unavailable then 3-factor PCC or fresh frozen plasma (FFP dose range 10-20ml/kg) may be given. Vitamin K will also be

administered but should not be relied upon for rapid correction of factor deficiency. Recombinant activated factor VII (rFVIIa) should not be given in isolation to reverse VKA-related coagulopathy. Therapy will target an INR < 1.3 within 4 hours of arrival.

Subjects with ICH during concomitant use of heparin should have heparin withheld and be treated with appropriately dosed protamine sulfate.

Subjects with a history of using antiplatelet medications (i.e. aspirin, clopidogrel etc.) do not routinely require platelet transfusion, but should have antiplatelet therapy withheld (PATCH Trial, 2016). However, subjects randomized to surgery, or those that require a surgical intervention as part of routine medical management, may be considered for platelet transfusion as determined by the treating physician. An intravenous dose of 1-deamino-8-D- arginine vasopressin (DDAVP), 0.4mcg/kg IV, may be considered in the treatment of dysfunctional platelets. Subjects that have thrombocytopenia with a platelet count < 75,000/mcL will receive platelet transfusion.

Direct oral anticoagulants (DOAC) often do not have widely available tests of activity or specific reversal agents; therefore, detection and reversal of these should follow routine local care. In general, a normal thrombin time and activated partial thromboplastin time likely excludes clinically relevant levels of dabigitran. Dabigitran reversal will be performed with idarucizumab, the FDA approved reversal agent for dabigitran. If idarucizumab is unavailable, then reversal may follow local standard.

The remaining DOACs do not currently have specific reversal agents and often routine clinical testing does not reliably assess coagulopathy. Reversal of DOACS for which no antidote exists may follow local standard. However, given challenges of confirming response to reversal these patients should be enrolled and randomized only if the investigator is confident that no clinically significant coagulopathy exists.

During the first 72 hours of care, INR will be maintained < 1.3 and Platelets will be kept > 75,000/mcL. Monitoring for coagulopathy, thrombocytopenia, or platelet dysfunction during the ICU course may follow routine clinical care.

Anemia – Anemia is frequently observed in the course of routine acute care. Packed Red Blood Cells (PRBC) should be transfused for hemoglobin < 7gm/dL. Subjects that develop acute blood loss anemia will be treated per local standards, as directed by the attending physician, without specific hemoglobin goals during the resuscitation period.

Deep Venous Thrombosis/Pulmonary Embolism Prophylaxis and Treatment – Subjects will be treated with intermittent pneumatic compression of the lower extremities beginning on Hospital Day 0, unless contraindicated based on clinical determination. Chemoprophylaxis with subcutaneous heparin (low- molecular weight heparin (LMWH) may be used) for DVT prevention should begin only after confirming clinical and radiographic hemorrhage stability. If the hemorrhage is clinically stable, chemoprophylaxis will commence within 4 days of admission or completion of a surgical procedure.

Systemic anticoagulation or inferior vena cava (IVC) filter placement is indicated for subjects that develop symptomatic DVT or PE. Subjects that develop symptomatic thromboembolism within 48 hours from admission or MIPS should receive an IVC filter. Beyond the first 48 hours following admission or surgery, the treating team may choose between these two treatment options based on the clinical situation. Heparin dose and clinical therapeutic targets may follow local standards for the treatment of DVT/PE in the setting of ICH. If the patient develops hemodynamic instability related to thromboembolism treatment decisions are left to the treating team.

Glucose Management – Glucose should be monitored per local practice to target euglycemia. Both hypo- and hyperglycemia should be avoided and blood glucose should be maintained between 80 – 180 mg/dL.

Temperature Management – Fever should be avoided. When a fever (Temp > 38.5°C) is identified it is necessary to investigate and treat the underlying etiology. Temperature may be managed with antipyretics (i.e. acetaminophen), cooling blanket, ice packs, or one of the marketed temperature control systems. Shivering related to temperature modulation will be managed according to local protocols to maintain a Bedside Shivering Assessment Scale (BSAS) of 0-1 at all times. Targeted mild hypothermia will not be routinely used unless treating intracranial hypertension.

Seizure Prophylaxis – Subjects with cortical ICH, those randomized to MIPS, or those that receive any neurosurgical procedure will receive a minimum effective dose of levitiracetam for seizure prophylaxis for 7 days. Subjects with subcortical ICH may be considered for seizure prophylaxis at the discretion of the attending physician. Phenytoin is not recommended for seizure prophylaxis.

Continuous electroencephalography (cEEG) should be considered if a subject's exam is worse than is expected based on the primary injury, if there is clinical deterioration without clear explanation, or if a seizure is appreciated during the course of care. Clinically significant seizures and status epilepticus require rapid and aggressive treatment. AEDs may be initiated, titrated, and continued per local routine care after a seizure is identified.

Intracranial Pressure Monitoring and Treatment – Subjects that are comatose, defined as a GCS < 8, over a period of at least 6 hours and with neuroimaging evidence of elevated ICP,

or those with obstructive hydrocephalus should be considered for ICP monitoring with, or without, CSF diversion, when appropriate. Monitoring device selection is left to the discretion of the treating clinician. ICP is considered elevated when it is > 20mmHg for > 15 minutes. Treatment goals are ICP < 20mmHg and CPP > 60mmHg.

General efforts to limit ICP elevations should be made during routine care of the ICH subject. The use of adequate sedation and analgesia is recommended for anxiolysis, pain control, and when appropriate, to optimize ventilator tolerance. Head of bed should be maintained at around 30° with the neck aligned in a neutral position. Fever should be treated as detailed in the Temperature Management section. Normal sodium levels (135 – 145 meq/L) should be targeted with avoidance and correction of hyponatremia (serum Na < 135 meq/L).

When general efforts to control ICP are ineffective then treatment of elevated ICP should be conducted progressively: 1. If available, perform CSF diversion with ventricular drainage. 2. Administer hyperosmolar therapy with either hypertonic saline or mannitol. We recommend hypertonic saline when available. Doses may be repeated per local practice. Hypertonic saline therapy should be stopped if serum Na > 160 meq/L, or plasma osmolarity > 360 mOsm/L. 3. Neuromuscular blockade may be considered. 4. Mild hyperventilation may be induced, with a goal PaCO2 of 30- 35mmHg, while making efforts toward more definitive care.

If the above therapies fail to bring the ICP into goal target range, then the treating clinician may consider metabolic suppression with deep sedation (recommend titration of sedative to burst suppression on cEEG), or mild hypothermia (32-34°C) for ICP control. DHC may be considered for treatment of refractory intracranial hypertension. The use of corticosteroids for ICP control is prohibited.

IVH Treatment (obstructive hydrocephalus) – Obstructive hydrocephalus should be treated with CSF diversion using ventricular catheters as directed by the treating team. Intrathecal tPA

may be considered on a per-subject basis by the treating team only after confirming hemorrhage and clinical stability, between 24-72 hours of hemorrhage or surgery.

Ventricular catheter weaning may follow local protocols. Ventriculoperitoneal shunts should be utilized as necessary.

Other Surgery (DHC, Lobar hematoma evacuation) – Subjects that are randomized to standard therapy may receive neurosurgical procedures as would be routinely performed outside of this clinical trial. Surgical hematoma evacuation may be performed, using standard non-investigational surgical techniques, if the investigator and treating team believe that it is likely to benefit the subject and represents reasonable routine care. The decision to perform decompressive hemicraniectomy (DHC) may be applied to either treatment arm with poor mental status, significant midline shift, large hematomas, or refractory elevation in ICP.

Nutritional Support – Nutritional support will be initiated as soon as possible after admission with caloric needs being met by the least invasive means possible. Dysphagia screening, or a formal swallow evaluation, should be performed before permitting oral nutrition. If oral nutrition is not clinically appropriate within 24 hours of admission, enteral access will be obtained and tube feedings will begin. Full caloric support (30kcal/kg with 1.5gm protein/kg) will be met within 72 hours of admission unless contraindicated by the clinical situation.

Respiratory Care – Maintain pulse oximetry (SpO_2) > 90%, or arterial partial pressure of oxygen (PaO_2) > 60mmHg at all times. When possible partial pressure of CO_2 will initially be maintained between 35 – 45mmHg using standard lung protective strategies to minimize risk of lung injury. Hypercarbia ($PaCO_2$ > 45mmHg) should be carefully avoided when possible. During mechanical ventilation, and when clinically appropriate, it is recommended that daily

spontaneous breathing trials (SBT) be performed to determine if the patient may be liberated from mechanical ventilation. SBTs may be performed per local routine with determination of potential for ventilator independence made by the treating clinician. Tracheostomy should be considered by day 14 if ventilator liberation has been unsuccessful.

Withdrawal of Technological Support – Aggressive care will be continued for a minimum of 72 hours, with postponement of do-not-resuscitate (DNR) orders for a minimum of 48 hours from admission. Those that have pre-existing DNR orders, or have an advanced directive that would reasonably prohibit aggressive care, are excluded from entry into the trial.
