# Supplementary material for: Early Minimally Invasive Removal of Intracerebral Hemorrhage (ENRICH): Study protocol for a multi-centered two-arm randomized adaptive trial
Source: Front Neurol. 2023 Mar 16;14:1126958. doi: 10.3389/fneur.2023.1126958 (PMC10061000; doi:10.3389/fneur.2023.1126958)

ENRICH Adaptive Design Report

Prepared by Ben Saville, Ph.D.
Berry Consultants

October 13, 2016

Contents

1 Introduction **3**
1.1 Clinical Study Objective 3
1.2 Primary Endpoint 3
1.3 Key Secondary Endpoints 3

2 Statistical Modeling **4**
2.1 Model 4
2.2 Error Variance 5
2.3 Longitudinal Model 5
2.4 Evaluation of Posterior Estimates 7
2.5 Conventions for Missing Data 7

3 Study Design **7**
3.1 Allocation 7
3.2 Timing of Trial Updates 7
3.3 Futility Stopping and/or Enrichment Criteria 7
3.4 Predicted Success Criteria 8
3.5 Final Evaluation Criteria 8
3.6 Study Flowchart 9

4 Simulation Scenarios **9**
4.1 Accrual Profiles 9
4.2 Dropout Profiles 10
4.3 Underlying Control Distribution 11
4.4 Treatment Effect Profiles 11
4.5 Localized Treatment Effects 11
4.6 Longitudinal Correlation 11
4.7 Prevalence of Locations 12
4.8 Simulating Patients 12

5 Operating Characteristics **12**
5.1 Expected Control Distribution, Proportional Effect 13
5.2 Worst Case Control Distribution 16
5.3 Best Case Control Distribution 17
5.4 Expected Control Distribution, Localized Benefit 1-3 18
5.5 Expected Control Distribution, Localized Benefit 4-6 19
5.6 Simulation Summary 20
5.7 Computational Details 20

6 Example Trials **20**
6.1 Example 1 . . . 21
6.1.1 Example 1: Update 1 . . . 22
6.1.2 Example 1: Update 2 . . . 23
6.1.3 Example 1: Final . . . 24
6.2 Example 2 . . . 25
6.2.1 Example 2: Update 1 . . . 26
6.2.2 Example 2: Final . . . 27
6.3 Example 3 . . . 28
6.3.1 Example 3: Update 1 . . . 29
6.3.2 Example 3: Update 2 . . . 30
6.3.3 Example 3: Update 3 . . . 31
6.3.4 Example 3: Update 4 . . . 32
6.3.5 Example 3: Update 5 . . . 33
6.3.6 Example 3: Final . . . 34
6.4 Example 4 . . . 35
6.4.1 Example 4: Update 1 . . . 36
6.4.2 Example 4: Update 2 . . . 37
6.4.3 Example 4: Update 3 . . . 38
6.4.4 Example 4: Update 4 . . . 39
6.4.5 Example 4: Update 5 . . . 40
6.4.6 Example 4: Update 6 . . . 41
6.4.7 Example 4: Update 7 . . . 42
6.4.8 Example 4: Final . . . 43

1 Introduction

The ENRICH trial is an adaptive, prospective, IDE exempt, post-market, randomized, multi-center study of early (<24 hours) minimally invasive parafascicular approach for intracerebral hemorrhage evacuation, to evaluate mortality, morbidity, and clinical outcomes against medical treatment. The design incorporates an adaptive sample size between 150 and 300 patients, frequent interim analyses, and equal randomization between surgery and control. The study population includes patients with Intracranial Hemorrhage (ICH) with hemorrhage volumes between 30-80 cc's, in either Anterior Basal Ganglia (ABG) or Lobar locations.

1.1 Clinical Study Objective

The goal of this study is to test whether the surgical approach can improve 180-day functional outcomes, based on utility weighted modified-Rankin Score (mRS), in patients with ICH compared to standard medical management.

1.2 Primary Endpoint

The primary efficacy endpoint is the mRS utility weighted scale at 180 days post randomization, as shown in Table 1. We will specifically test the following hypotheses:

$H_0 : \mu_T \leq \mu_C$ versus $H_A : \mu_T > \mu_C$

where μ_T and μ_C are the mean mRS utility for patients in the treatment and control groups, respectively. If the Bayesian posterior probability that μ_T is greater than μ_C, denoted by $\Pr(\mu_T > \mu_C)$, is greater than 0.975, then the study will demonstrate superiority of the surgery versus control.

Table 1: Distribution of mRS Values in Control Population

mRS	Utility Weights
0	1.0
1	0.91
2	0.76
3	0.65
4	0.33
5	0
6	0

1.3 Key Secondary Endpoints

Key secondary outcomes include but are not limited to the following:

1. Proportion of patients with mRS equal to 0,1,2,3: A test of proportions will be performed using a one-sided $\alpha = 0.025$ to determine if the proportion is greater in the surgery group.
2. Ordinal mRS: A Wilcoxon Sum Rank test (one-sided $\alpha = 0.025$) will be used to test whether the distribution of mRS scores is less than (i.e. more favorable) for the surgery group compared to control.
3. All primary and key secondary analyses will be repeated for two distinct subsets of patients: 1) patients presenting to hospital in 8 hours or less since onset of symptoms; and 2) patients presenting to hospital in 12 hours or less since onset of symptoms.
4. Other secondary endpoints: Length of stay, 90 day outcomes, economic outcomes, etc.

A comprehensive list of secondary endpoints will be included in the Statistical Analysis Plan (SAP).

2 Statistical Modeling

This section describes the statistical modeling used in the design. The modeling is Bayesian in nature. Let Y_i be the primary outcome (utility weighted mRS) measured at 6 months for the ith subject. Higher response corresponds to subject improvement. We model the outcomes as

$$Y \sim \mathrm{N}(\mu_{g,d}, \sigma^2)$$

where $\mu_{g,d}$ is the underlying mean response for arm d ($d = 0$ for control and $d = 1$ for the experimental arm) in location g ($g = 1$ for ABG and $g = 2$ for Lobar). We define

$$\mu_{g,0} = \gamma_g$$

for the control arm and

$$\mu_{g,1} = \gamma_g + \theta_g$$

for the experimental arm. Thus, the parameter θ_g represents the effect of the experimental treatment relative to control.

2.1 Model

A hierarchical model is used to borrow information across locations on the control arm. The model is:

$$\gamma_g \sim N(\mu_\gamma, \tau_\gamma^2),$$

with hyperpriors:

$$\mu_\gamma \sim N(0, 1^2),$$

and

$$\tau_\gamma^2 \sim IG(0.5, 0.005),$$

where IG(a, b) is the inverse gamma distribution defined by:

$$f(x|a, b) = \frac{b^a e^{-b/x}}{x^{a+1}\Gamma(a)}.$$

A hierarchical model is also used to borrow information across locations on the treatment effect. The model is:

$$\theta_g \sim N(\mu_\theta, \tau_\theta^2),$$

with hyperpriors:

$$\mu_\theta \sim N(0, 1^2),$$

and

$$\tau_\theta^2 \sim IG(0.5, 0.005),$$

Each hierarchical model allows dynamic borrowing of information between locations such that more borrowing occurs when the locations are consistent and less borrowing occurs when the locations differ. In this way, the model is a compromise between the two alternate extremes of either a completely pooled analysis or a separate analysis in each location. The variance components τ_γ and τ_θ are the key parameter that controls the degree of borrowing among locations. Small values of τ_γ or τ_θ result in a greater degree of borrowing while large values of τ_γ or τ_θ correspond to less borrowing. The parameters τ_γ and τ_θ are estimated using the data, so that the observed between-location variation is a key component of the model behavior.

In addition to the model described above, we also fit an "across locations" model that estimates a single parameter for the treatment effect (ignoring location classification). This model is used for the primary endpoint at the final decisive analysis. The model is:

$$\theta \sim N(0, 100^2).$$

This model assumes a constant treatment effect across locations, but the control rate may still differ among locations.

2.2 Error Variance

The error variance (across locations and arms) is centered at 0.36^2 (obtained from expected control mRS distribution) with a weight of 1 via the model:

$$\sigma^2 \sim IG(0.5, 0.0648).$$

2.3 Longitudinal Model

In addition to the final endpoint at 6 months, subjects will have scheduled data assessments at 3 months. At any trial update, there may be subjects in each of the following categories:

- Complete subjects, whose final endpoint value is known,
- Incomplete subjects with only intermediate visits but no final endpoint,
- Subjects with no data at all.

We construct a longitudinal model to allow the unobserved final endpoint to be imputed from the partial data. Let Y_i be the final endpoint value for subject i and let y_{it} be the intermediate response at visit t. We fit a set of models (one per visit) that pools data across locations and arms.

The Kernel density model is a non-parametric re-sampling approach that is ideal for circumstances where the relationship between the trial update time and the final endpoint is not known or not canonical. In this method, existing subjects with known final endpoint data are selected based on how close their intermediate visit data is to that of the subject whose final endpoint is to be imputed. This proximity uses a Normal kernel to derive the weighting, with a bandwidth (h_x) derived dynamically. The imputed value is then sampled from a Normal distribution centered on the selected subjects final endpoint, again using a dynamically adjusted bandwidth (h_y). The probability of selecting a subject when sampling is determined using a Normal probability density centered on the observed update endpoint value of the subject whose final endpoint we wish to impute. The Normal probability density has standard deviation h_x. The value imputed for the subject is sampled from a Normal distribution centered on the selected subjects final endpoint value with standard deviation h_y. Weak initial values for h_x and h_y would be the expected standard deviation of the observations at the visit and the expected final standard deviation of the endpoint observations. Smaller h_x would more strongly select values of X close to the observed update for which a final value is being imputed. Smaller h_y would reflect an expected conditional variance of the final visit given the update value. The parameters are:

- Initial value for h_y, the noise added to the sampled endpoint value
- Kernel minimum number of subjects: the minimum number of subjects in the model who must have final endpoint data before the h_x and h_y are updated based on the observed variances and correlation
- Initial value for h_x, the width of the normal kernel used for sampling.

The procedure is as follows. Assume a trial update value, at time j of Y_j. The T-month value for the subject is unknown and thus a value of Y_T is to be imputed. Let $(X_1, Z_1), \ldots, (X_n, Z_n)$ be the set of values for the previous subjects for whom there exists a trial update value X_k and final value Z_T. To impute a value of Y_T for a subject with update value Y_j, a pair (X_k, Z_k) is selected with probability:

$$\frac{\exp(-\frac{1}{2h_x^2}(Y_j - X_k)^2)}{W_p + \sum_{k=1}^{n} \exp(-\frac{1}{2h_x^2}(Y_j - X_k)^2)}.$$

For the value of X_k that is selected, a value of Y_T is imputed from the following distribution:

$$Y_T \sim N(Z_k, h_y^2).$$

The extra W_p in the denominator of the probability of selecting each pair is for the prior observation of (Y_j, Y_j) that is added to the data for the kernel. The W_p is the weight given to the prior observation of (Y_j, Y_j). Thus, with probability:

$$\frac{W_p}{W_p + \sum_{k=1}^{n} \exp(-\frac{1}{2h_x^2}(Y_j - X_k)^2)},$$

an observation of Y_T is selected from the distribution:

$$Y_T \sim N(Y_j, h_y^2).$$

The bandwidths, h_x and h_y are selected based on the criterion:

$$h_i^* = \sigma_i(1-\rho^2)^{5/12}(1+\rho^2/2)^{-1/6}n^{-1/6}, \qquad i = x, y$$

where

- σ_x is the standard deviation of the observed responses at time j $(X_1, \ldots, X_n)$
- σ_y is the standard deviation of the observed final responses $(Z_i, \ldots, Z_T)$
- ρ is the correlation coefficient between X and Z.

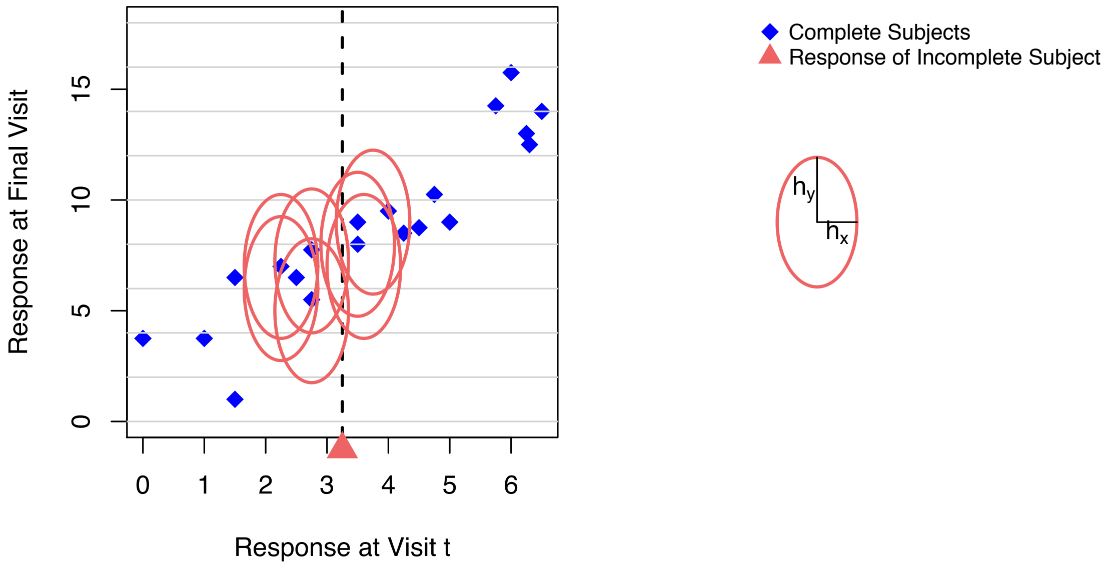

Figure 1: Illustration of the kernel density longitudinal model

The parameters for the model are shown in Table 2.

Table 2: Prior parameters for the longitudinal model

Kernel bandwidth h_y	Kernel minimum subjects	SD of prior mean h_x
2	6	2

2.4 Evaluation of Posterior Estimates

The Bayesian model fitted to the data at each update contains a final endpoint model and a longitudinal model. The posterior is calculated as:

$$p(\omega|Y) \propto \prod_{i=1}^{n} p(y_i|\varphi)p(\varphi) \prod_{i=1}^{n}\prod_{t=1}^{L} p(y_{it}|\psi)p(\psi)$$

where φ is the set of parameters for the final endpoint model, $p(\varphi)$ is the prior for those parameters, ψ is the set of parameters for the longitudinal model, $p(\psi)$ is the prior for those parameters, y_i is the final response for each subject, y_{it} is the response for each subject at each visit, n is the number of subjects, and L is the number of visits. The posterior is evaluated using MCMC with individual parameters updated by Metropolis Hastings (or Gibbs sampling where possible), using only the y_i and y_{it} data available at the time of the update.

2.5 Conventions for Missing Data

At any analysis, some subjects may have missing data for the final endpoint. The missing data could result from the subject dropping out of the study, or because the subject simply has not yet reached the final visit.

If the subject has not yet reached the final visit, but has data from an intermediate visit then the missing endpoint is "imputed" from the longitudinal model. Multiple imputation involves sampling, in each MCMC iteration, from the posterior estimate of the subjects final endpoint given their intermediate visit data and the longitudinal model and the current longitudinal parameters. Thus the imputed value has the uncertainty of the longitudinal model and the estimates of its parameters. If the subject has no update visit results, the subjects endpoint value is simply imputed from the estimate of the location response for the subjects treatment arm (effectively contributing no information to the update of that estimate.)For any subject whose final endpoint is unknown due to drop out, the final outcome will be multiply imputed from the Bayesian model as above.

3 Study Design

3.1 Allocation

The trial will enroll up to a maximum of 300 subjects with a hemorrhage in either the anterior basal ganglia (ABG) or the lobar location. Patients will be randomized to surgery or control in a 1:1 ratio. Randomization will be stratified by location and Index GSC (< 9 or ≥ 9), with random block sizes of 2, 4, and 6. No restrictions are placed on the number of subjects that may be enrolled per location.

3.2 Timing of Trial Updates

Trial updates will occur after 150, 175, 200, 225, 250, and 275 subjects enrolled. At each trial update, the data will be evaluated to determine whether one or more locations should stop accrual with the current sample size.

3.3 Futility Stopping and/or Enrichment Criteria

As noted previously, θ_g is the mean difference between surgery and control groups within location g for the mRS utility scale. Let $Pr(\theta_g > 0.075)$ be the posterior probability that the mean mRS utility is greater in

the surgery group than controls by at least 0.075 within location g. At a given trial update, if the following condition is met

$$Pr(\theta_g > 0.075) < 0.20,$$

accrual will be stopped within group g due to a low probability of a meaningful difference between surgery and control, provided at least 30 patients have complete 180-day outcomes within the location. If neither location has at least 30 patients with complete 180-day outcomes at a scheduled trial update, the trial update will be skipped.

3.4 Predicted Success Criteria

As noted previously, θ is the overall mean difference between surgery and control (pooling 2 locations) for the mRS utility scale. Let $Pr(\theta > 0)$ be the posterior probability that the mean mRS utility is greater in the surgery group than controls in the pooled population. If the following condition is met

$$Pr(\theta > 0) > 0.99,$$

accrual will be stopped to all remaining locations due to predicted success, provided at least 60 patients have complete 180-day outcomes. If the trial stops for predicted success or reaches the pre-determined maximum sample size of $n = 300$, a final decisive analysis is performed 180 days after the last patient is enrolled.

3.5 Final Evaluation Criteria

Provided the trial has not stopped for futility, a final decisive analysis will be conducted 180 days after the last patient is enrolled. The study will demonstrate superiority of the surgical intervention compared to control if the condition

$$Pr(\theta > 0) > 0.975$$

is met in the pooled (across locations) analysis. This test will include all patients enrolled regardless of whether enrichment has taken place. If no enrichment has occurred (neither location stopping for futility) and the final evaluation criteria is met, superiority will be claimed for both ABG and Lobar locations.

If enrichment occurs (futility rule met in one of the two locations) at any point in the trial, and the final evaluation criteria is met, superiority is only claimed for the location that did not meet the futility criteria, despite the final evaluation criteria including patients from both locations.

A key attribute of the design is that although the final sample size may be 150, 175, 200, 225, 250, 275, or 300, there is only one final decisive analysis to evaluate the superiority of the surgery, which occurs 180 days after the last patient is enrolled.

3.6 Study Flowchart

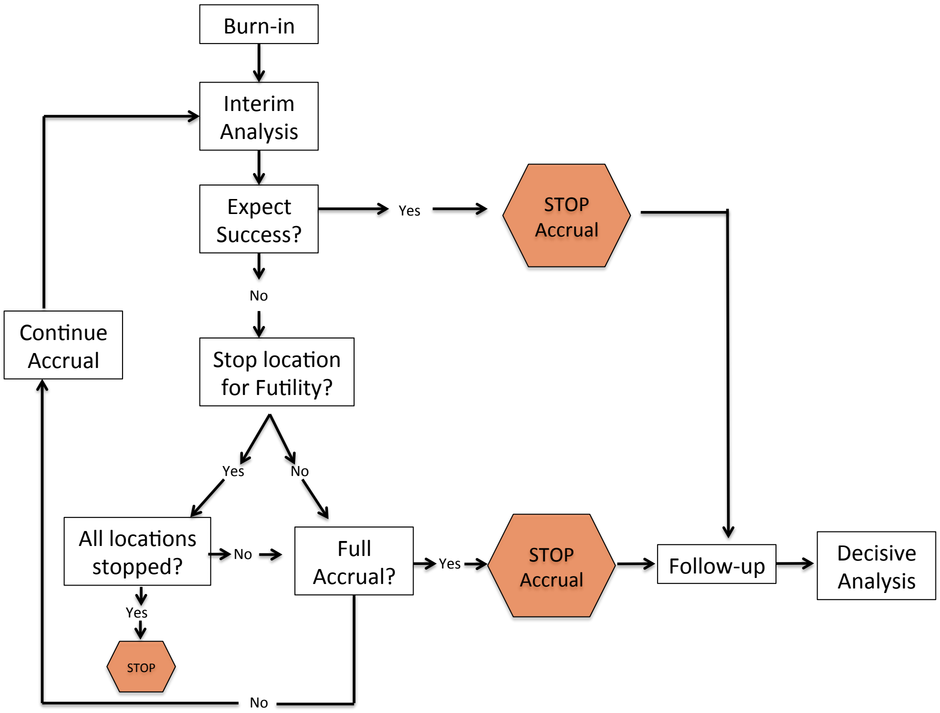

4 Simulation Scenarios

Simulations are created using a variety of assumptions for accrual rate, dropout rate, mRS control distribution, treatment effect, longitudinal correlation, and prevalence of ABG/Lobar locations.

4.1 Accrual Profiles

Subject accrual to the trial is simulated from a Poisson process. As subjects become available, their location membership is simulated based on the prevalence of the group within the population. Three accrual profiles are used, corresponding to a maximum enrollment rate of 5, 15, or 30 patients per month. All three profiles assume a 6 month ramp-up to achieve the maximum accrual.

The profiles are also shown in Figure 2. Note that if any group stops accrual for any reason, then the overall accrual rate will be reduced in proportion to the prevalence of the discontinued group.

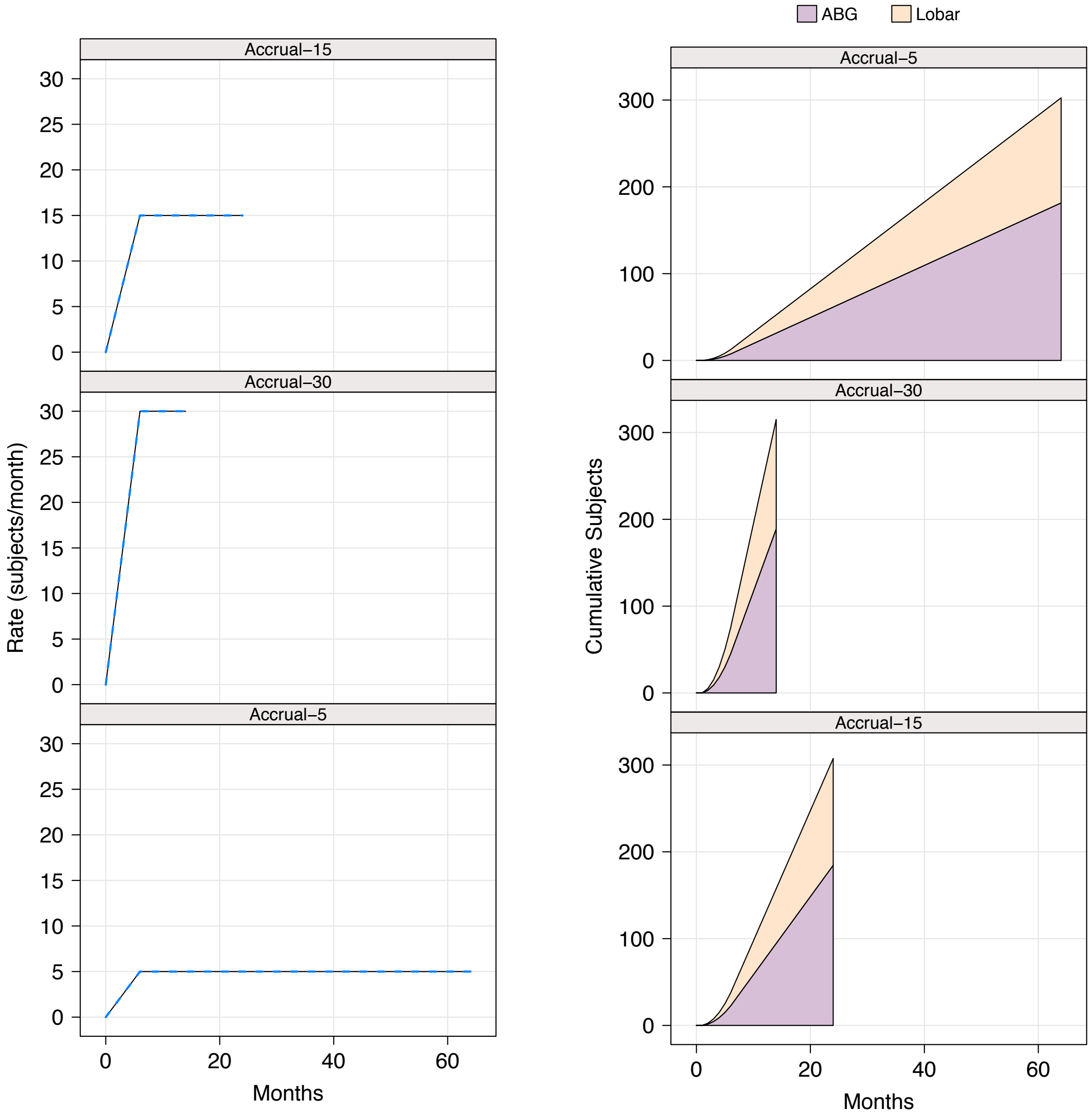

Figure 2: Accrual Profiles. The right side of the figure shows the expected rate of accrual over time. There is one panel per accrual scenario. The left side of the figure shows the expected cumulative number of subjects enrolled over time. The area under the curve is shaded according to location membership.

4.2 Dropout Profiles

We simulate data with two dropout profiles:

1. No dropout in either treatment group
2. 10% random dropout in each treatment group. This overall dropout rate consists of 5% dropout between 0 and 3 months, and 5% dropout between 3 and 6 months.

4.3 Underlying Control Distribution

Assessment of design via simulation depends on assumed distribution of mRS values. Using historical studies, we have developed an "expected", "worst", and "best" mRS distribution for the control population.

Table 3: Sensitivity to mRS Control Distribution

	Historical Proportions			BrainPath			
mRS	STICH-II	MISTIE-II	Austria	Worst	Expected	Best	Weights
0	6%	0%	0%	1%	10%	20%	1.0
1	20%	2.5%	0%	2%	21%	30%	0.91
2	14%	5%	10%	7%	23%	22%	0.76
3	11%	5%	8%	7%	12%	9%	0.65
4	11%		9%	9%	10%	6%	0.33
5	14%	80%*	4%	4%	9%	5%	0
6	24%		70%	70%	15%	7%	0

* 80% for mRS 4-6 in MISTIE-II

4.4 Treatment Effect Profiles

Simulated treatment effects will be defined on the mRS utility scale in addition to the mRS proportion scale. Let $\Delta = p_T - p_C$ be the difference in proportion of patients with mRS ≤ 3 for treatment versus control. A proportional odds logistic modeling strategy is used to shift the mRS distribution in the treatment study population, such that the treatment benefit is proportional across all mRS values (i.e. the treatment is equally beneficial to patients no matter their underlying mRS status). The model generating the treatment group distribution is

$$\text{logit}\{\Pr(Y_i \geq j)\} = \alpha_j + \beta x_i,$$

where Y_i denotes the mRS value for patient i, α_j denotes the log odds of mRS $\leq j$ if patient i is in the control group, x_i is an indicator function that takes the value 1 if patient i is in the treatment group and 0 otherwise, and β is the increment in the log odds of mRS $\leq j$ for a patient in the treatment group relative to the control group. The corresponding probability that patient i has mRS $\leq j$ is given by:

$$p_{ij} = \frac{\exp(\alpha_j + \beta x_i)}{1 + \exp(\alpha_j + \beta x_i)}$$

The α_j parameters are determined by the assumed distribution of the control distribution. Using the above model, we define small, medium, and large treatment effects by setting $\beta = 2.3$, 2.9, and 3.5, respectively.

4.5 Localized Treatment Effects

In addition to the proportional effect, we also evaluate "localized" treatment effects, in which the surgery is only beneficial to patients with a specific range of mRS values. As with the proportional effect, a proportional odds modeling strategy is used but the effect is limited to a narrow range of patients. Specifically, we investigate a treatment effect in patients with mRS equal to 1, 2, and 3 (0-6 mRS scale), or localized to mRS of 4, 5, and 6.

4.6 Longitudinal Correlation

It is assumed that the correlation between 3 month and 6 month mRS utility weighted scores is equal to either 0 (no correlation) or 0.4 (moderate correlation).

4.7 Prevalence of Locations

We use prevalence rates for locations of either 60% ABG/40% Lobar, or 80% ABG/20% Lobar. Alternative scenarios in which Lobar is more prevalent than ABG can be obtained by switching the labels of ABG and Lobar, and thus do not require additional simulations.

4.8 Simulating Patients

Given the above assumptions, patient responses are simulated from a Normal (continuous) distribution with a mean and standard deviation corresponding to the appropriate mRS distribution for a given scenario.

5 Operating Characteristics

Results are summarized for various combinations of the assumed parameters (accrual rate, dropout rate, mRS control distribution, treatment effect profile, longitudinal correlation, prevalence of ABG/Lobar locations). Results are summarized below with the following information:

- Acc: Assumed accrual rate (monthly)
- Drop: Assumed dropout rate (%)
- Long: Assumed longitudinal correlation
- Δ_1: Assumed treatment effect for ABG location
- Δ_2: Assumed treatment effect for Lobar location
- Avg Sample Size: Average sample size for overall study, and separately by 1) ABG or 2) Lobar location
- Months: Average total study length in months
- Power: Probability of claiming treatment benefit in at least one location
- StopPredSucc: Probability of stopping early for predicted success and either 1) winning the trial (significant benefit), or 2) losing the trial (no significant benefit)
- Stop Futility: Probability of stopping early for futility
- StopMax: Probability of enrolling to the maximum sample size (N=300) and either 1) winning the trial, or 2) losing the trial
- Successful Indications: Probability of claiming success for either 1) ABG alone, 2) Lobar alone, or 3) Both ABG and Lobar locations

5.1 Expected Control Distribution, Proportional Effect

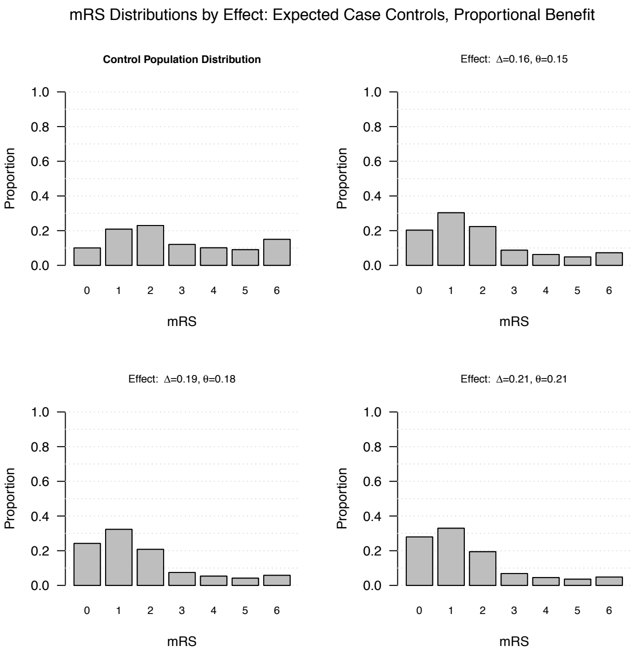

Table 4: mRS Distributions by Effect: Expected Case Controls

	Δ	θ	mRS Proportion 0	1	2	3	4	5	6
Control	0.16	0.15	0.10	0.21	0.23	0.12	0.10	0.09	0.15
Small	0.16	0.15	0.20	0.30	0.22	0.09	0.06	0.05	0.07
Medium	0.19	0.18	0.24	0.32	0.21	0.08	0.05	0.04	0.06
Large	0.21	0.21	0.28	0.33	0.19	0.07	0.05	0.03	0.05

Table 5: ABG/Lobar=(0.60,0.40): Drop=0%; Long=0

Parameters Acc	Drop	Long	Effect Size Δ_1	Δ_2	Avg Sample Size Overall	N_1	N_2	Months	Power	StopPredSucc Win	Lose	Stop Futility	StopMax Win	Lose	Successful Indications ABG	Lobar	Both
5	0	0.0	0	0	203	117	87	54.7	0.034	0.020	0.002	0.818	0.014	0.146	0.004	0.002	0.028
			small	small	191	115	76	46.0	0.976	0.927	0.003	0.009	0.049	0.012	0.032	0.022	0.922
			med	med	171	103	68	41.2	0.994	0.983	0.003	0.000	0.011	0.003	0.013	0.008	0.973
			small	0	244	168	76	63.1	0.780	0.610	0.014	0.058	0.170	0.148	0.422	0.000	0.358
			med	small	179	108	71	42.9	0.993	0.964	0.002	0.000	0.029	0.005	0.033	0.008	0.952
			large	small	168	102	66	40.5	0.998	0.990	0.002	0.000	0.008	0.000	0.029	0.000	0.969
			large	0	207	138	69	52.8	0.970	0.923	0.007	0.006	0.047	0.017	0.456	0.000	0.514
			0	small	262	114	148	80.9	0.626	0.422	0.006	0.107	0.204	0.261	0.001	0.413	0.212
			0	med	255	112	143	78.2	0.793	0.617	0.006	0.046	0.176	0.155	0.001	0.532	0.260
15			0	0	223	120	103	24.8	0.024	0.004	0.001	0.799	0.020	0.175	0.004	0.005	0.015
			small	small	249	146	103	24.0	0.959	0.752	0.002	0.021	0.207	0.014	0.037	0.078	0.844
			med	med	228	136	92	22.1	0.984	0.889	0.003	0.007	0.095	0.004	0.017	0.031	0.936
			small	0	270	176	94	26.9	0.679	0.338	0.007	0.146	0.341	0.167	0.364	0.010	0.305
			med	small	236	141	95	22.7	0.981	0.840	0.002	0.013	0.141	0.003	0.041	0.038	0.902
			large	small	225	135	90	21.8	0.993	0.922	0.002	0.002	0.071	0.001	0.046	0.017	0.930
			large	0	261	171	90	25.8	0.931	0.660	0.006	0.033	0.271	0.027	0.476	0.002	0.453
			0	small	277	123	154	32.3	0.579	0.256	0.004	0.125	0.323	0.290	0.002	0.421	0.156
			0	med	278	123	155	32.3	0.767	0.388	0.007	0.059	0.379	0.167	0.001	0.571	0.195
30			0	0	278	155	123	16.9	0.022	0.001	0.000	0.667	0.021	0.305	0.004	0.010	0.008
			small	small	297	176	122	16.7	0.952	0.163	0.000	0.015	0.789	0.024	0.052	0.088	0.812
			med	med	295	175	120	16.5	0.984	0.293	0.000	0.000	0.691	0.004	0.027	0.051	0.906
			small	0	295	179	116	16.8	0.629	0.026	0.000	0.129	0.603	0.226	0.245	0.018	0.366
			med	small	296	176	120	16.6	0.977	0.228	0.000	0.004	0.749	0.009	0.056	0.054	0.867
			large	small	294	176	119	16.5	0.989	0.331	0.000	0.002	0.658	0.001	0.051	0.033	0.905
			large	0	298	182	116	16.8	0.883	0.072	0.002	0.035	0.811	0.062	0.365	0.005	0.513
			0	small	295	159	137	17.9	0.432	0.023	0.000	0.125	0.409	0.435	0.002	0.263	0.167
			0	med	297	160	138	18.0	0.583	0.033	0.000	0.063	0.550	0.347	0.001	0.349	0.233

* Group numbers correspond to 1) Anterior Basal Ganglia; 2) Lobar

Table 6: ABG/Lobar=(0.60,0.40): Drop=0%; Long=0.4

Parameters			Effect Size		Avg Sample Size					StopPredSucc		Stop	StopMax		Successful Indications		
Acc	Drop	Long	Δ_1	Δ_2	Overall	N_1	N_2	Months	Power	Win	Lose	Futility	Win	Lose	ABG	Lobar	Both
5	0	0.4	0	0	205	117	88	55.4	0.027	0.018	0.000	0.815	0.009	0.158	0.005	0.003	0.019
			small	small	187	112	75	45.1	0.971	0.921	0.006	0.006	0.050	0.017	0.027	0.018	0.926
			med	med	168	101	67	40.6	0.990	0.982	0.005	0.000	0.008	0.005	0.009	0.006	0.975
			small	0	239	164	74	61.9	0.803	0.644	0.021	0.051	0.159	0.125	0.429	0.001	0.373
			med	small	174	105	69	41.9	0.986	0.968	0.003	0.001	0.018	0.010	0.023	0.005	0.958
			large	small	165	100	66	39.9	0.997	0.988	0.000	0.000	0.009	0.003	0.020	0.001	0.976
			large	0	202	134	68	51.3	0.978	0.936	0.008	0.005	0.042	0.009	0.453	0.000	0.525
			0	small	260	114	146	79.8	0.634	0.460	0.011	0.088	0.174	0.267	0.001	0.407	0.226
			0	med	252	113	139	76.3	0.781	0.623	0.011	0.044	0.158	0.164	0.001	0.505	0.275
15			0	0	222	120	102	24.6	0.023	0.010	0.002	0.782	0.013	0.193	0.003	0.004	0.016
			small	small	233	138	95	22.7	0.959	0.811	0.009	0.009	0.148	0.023	0.029	0.044	0.886
			med	med	210	125	84	20.7	0.991	0.940	0.005	0.002	0.051	0.002	0.014	0.015	0.962
			small	0	270	178	92	27.0	0.683	0.383	0.013	0.097	0.300	0.205	0.359	0.005	0.319
			med	small	219	131	88	21.4	0.986	0.909	0.003	0.005	0.077	0.006	0.032	0.016	0.938
			large	small	205	124	82	20.3	0.992	0.952	0.004	0.003	0.040	0.001	0.025	0.005	0.962
			large	0	249	164	85	24.9	0.947	0.769	0.006	0.014	0.178	0.032	0.475	0.000	0.472
			0	small	278	122	156	32.6	0.616	0.294	0.012	0.091	0.322	0.281	0.002	0.449	0.165
			0	med	276	121	154	32.2	0.784	0.467	0.014	0.038	0.317	0.164	0.000	0.577	0.207
30			0	0	278	156	123	16.9	0.016	0.000	0.000	0.642	0.016	0.339	0.003	0.004	0.009
			small	small	290	173	117	16.3	0.965	0.384	0.000	0.010	0.581	0.023	0.026	0.033	0.906
			med	med	282	169	113	16.0	0.993	0.607	0.000	0.000	0.386	0.007	0.011	0.013	0.969
			small	0	296	181	115	16.8	0.657	0.076	0.003	0.073	0.581	0.266	0.264	0.002	0.391
			med	small	286	171	115	16.1	0.984	0.523	0.000	0.001	0.461	0.010	0.027	0.007	0.950
			large	small	279	168	112	15.9	0.994	0.641	0.000	0.000	0.353	0.006	0.024	0.006	0.964
			large	0	295	181	114	16.6	0.911	0.228	0.004	0.009	0.683	0.075	0.379	0.000	0.532
			0	small	297	159	138	18.0	0.443	0.026	0.002	0.072	0.417	0.482	0.001	0.250	0.192
			0	med	298	160	138	18.0	0.582	0.058	0.003	0.037	0.524	0.376	0.000	0.325	0.257

*Group numbers correspond to 1) Anterior Basal Ganglia; 2) Lobar

Table 7: ABG/Lobar=(0.60,0.40): Drop=10%; Long=0.4

Parameters			Effect Size		Avg Sample Size					StopPredSucc		Stop	StopMax		Successful Indications		
Acc	Drop	Long	Δ_1	Δ_2	Overall	N_1	N_2	Months	Power	Win	Lose	Futility	Win	Lose	ABG	Lobar	Both
5	10	0.4	0	0	209	119	90	57.1	0.023	0.008	0.004	0.789	0.015	0.184	0.001	0.004	0.018
			small	small	196	118	79	47.2	0.962	0.896	0.012	0.004	0.066	0.022	0.031	0.017	0.914
			med	med	173	104	69	41.6	0.992	0.979	0.003	0.002	0.013	0.003	0.016	0.005	0.971
			small	0	246	168	78	63.4	0.735	0.592	0.010	0.055	0.143	0.200	0.361	0.000	0.374
			med	small	182	109	72	43.5	0.987	0.965	0.005	0.002	0.022	0.006	0.026	0.006	0.955
			large	small	170	103	67	41.0	0.997	0.989	0.002	0.001	0.008	0.000	0.029	0.003	0.965
			large	0	211	140	70	53.4	0.966	0.899	0.007	0.006	0.067	0.021	0.424	0.000	0.542
			0	small	265	118	147	80.7	0.575	0.392	0.002	0.091	0.183	0.332	0.000	0.369	0.206
			0	med	258	115	143	78.2	0.727	0.557	0.012	0.046	0.170	0.215	0.000	0.468	0.259
15			0	0	227	123	104	24.9	0.018	0.004	0.002	0.784	0.014	0.196	0.003	0.002	0.013
			small	small	243	144	99	23.4	0.944	0.752	0.011	0.007	0.192	0.038	0.030	0.045	0.869
			med	med	218	130	88	21.2	0.991	0.917	0.001	0.004	0.074	0.003	0.010	0.014	0.967
			small	0	275	180	95	27.1	0.624	0.289	0.016	0.099	0.335	0.261	0.331	0.001	0.292
			med	small	228	137	91	21.9	0.972	0.867	0.007	0.008	0.105	0.013	0.028	0.012	0.932
			large	small	213	128	85	20.9	0.992	0.933	0.002	0.002	0.059	0.004	0.026	0.006	0.960
			large	0	259	170	90	25.5	0.912	0.663	0.012	0.016	0.249	0.059	0.441	0.000	0.471
			0	small	282	126	156	32.5	0.486	0.221	0.007	0.101	0.265	0.406	0.000	0.327	0.159
			0	med	281	126	155	32.4	0.675	0.333	0.009	0.043	0.342	0.273	0.000	0.465	0.210
30			0	0	282	158	124	17.0	0.023	0.002	0.000	0.616	0.021	0.359	0.004	0.006	0.013
			small	small	293	175	118	16.4	0.932	0.309	0.002	0.006	0.623	0.056	0.017	0.021	0.894
			med	med	286	171	115	16.1	0.984	0.500	0.000	0.000	0.484	0.014	0.009	0.011	0.964
			small	0	297	181	117	16.7	0.583	0.053	0.002	0.075	0.530	0.337	0.206	0.003	0.374
			med	small	289	173	116	16.2	0.975	0.427	0.001	0.002	0.548	0.019	0.021	0.010	0.944
			large	small	284	170	114	16.0	0.991	0.539	0.000	0.000	0.452	0.006	0.021	0.006	0.964
			large	0	296	181	115	16.6	0.856	0.175	0.000	0.011	0.681	0.132	0.309	0.001	0.546
			0	small	298	162	136	17.9	0.340	0.016	0.000	0.068	0.324	0.589	0.001	0.160	0.179
			0	med	298	162	136	17.9	0.473	0.036	0.000	0.038	0.437	0.489	0.000	0.243	0.230

*Group numbers correspond to 1) Anterior Basal Ganglia; 2) Lobar

Table 8: ABG/Lobar=(0.80,0.20): Drop=10%; Long=0.4

Parameters			Effect Size		Avg Sample Size					StopPredSucc		Stop	StopMax		Successful Indications		
Acc	Drop	Long	Δ_1	Δ_2	Overall	N_1	N_2	Months	Power	Win	Lose	Futility	Win	Lose	ABG	Lobar	Both
5	10	0.4	0	0	223	144	79	86.7	0.020	0.010	0.004	0.761	0.010	0.215	0.001	0.005	0.014
			small	small	199	157	42	49.2	0.935	0.875	0.014	0.013	0.060	0.038	0.014	0.019	0.902
			med	med	176	140	35	42.3	0.983	0.961	0.005	0.005	0.022	0.007	0.007	0.006	0.970
			small	0	226	184	43	54.2	0.819	0.694	0.020	0.056	0.125	0.105	0.200	0.000	0.619
			med	small	180	144	36	43.3	0.980	0.949	0.005	0.005	0.031	0.010	0.014	0.006	0.960
			large	small	167	134	33	40.3	0.997	0.986	0.002	0.000	0.011	0.001	0.018	0.002	0.977
			large	0	185	149	35	44.3	0.980	0.950	0.006	0.003	0.030	0.011	0.154	0.000	0.826
			0	small	278	148	130	137.2	0.399	0.234	0.007	0.117	0.165	0.477	0.002	0.327	0.070
			0	med	278	148	130	137.2	0.564	0.373	0.004	0.062	0.191	0.370	0.001	0.469	0.094
15			0	0	235	151	84	34.7	0.018	0.004	0.003	0.739	0.014	0.240	0.001	0.003	0.014
			small	small	243	190	53	24.3	0.945	0.755	0.005	0.008	0.190	0.042	0.013	0.042	0.890
			med	med	219	174	45	21.6	0.985	0.908	0.006	0.001	0.077	0.008	0.008	0.021	0.956
			small	0	261	206	55	25.4	0.778	0.476	0.010	0.076	0.302	0.134	0.165	0.002	0.611
			med	small	224	178	46	22.0	0.980	0.883	0.006	0.004	0.097	0.010	0.014	0.019	0.947
			large	small	207	165	42	20.4	0.997	0.958	0.000	0.000	0.039	0.003	0.013	0.007	0.977
			large	0	230	184	46	22.1	0.968	0.844	0.003	0.010	0.124	0.019	0.143	0.003	0.822
			0	small	283	158	125	48.4	0.338	0.161	0.004	0.125	0.177	0.533	0.000	0.267	0.071
			0	med	286	159	127	48.9	0.468	0.245	0.009	0.055	0.223	0.468	0.000	0.389	0.079
30			0	0	283	199	84	20.6	0.026	0.001	0.000	0.583	0.025	0.390	0.000	0.008	0.018
			small	small	292	232	60	16.5	0.958	0.306	0.002	0.004	0.652	0.036	0.001	0.029	0.928
			med	med	285	227	57	16.1	0.992	0.512	0.000	0.001	0.480	0.006	0.001	0.013	0.978
			small	0	296	235	61	16.7	0.807	0.130	0.002	0.032	0.677	0.157	0.011	0.009	0.787
			med	small	286	228	58	16.2	0.989	0.485	0.000	0.002	0.504	0.009	0.001	0.013	0.975
			large	small	278	223	56	15.8	0.998	0.650	0.000	0.000	0.348	0.002	0.001	0.009	0.988
			large	0	288	230	58	16.2	0.977	0.420	0.001	0.003	0.557	0.016	0.008	0.004	0.963
			0	small	298	203	95	22.4	0.174	0.021	0.000	0.066	0.153	0.760	0.000	0.113	0.061
			0	med	299	204	95	22.3	0.250	0.036	0.000	0.026	0.214	0.724	0.000	0.167	0.083

* Group numbers correspond to 1) Anterior Basal Ganglia; 2) Lobar

5.2 Worst Case Control Distribution

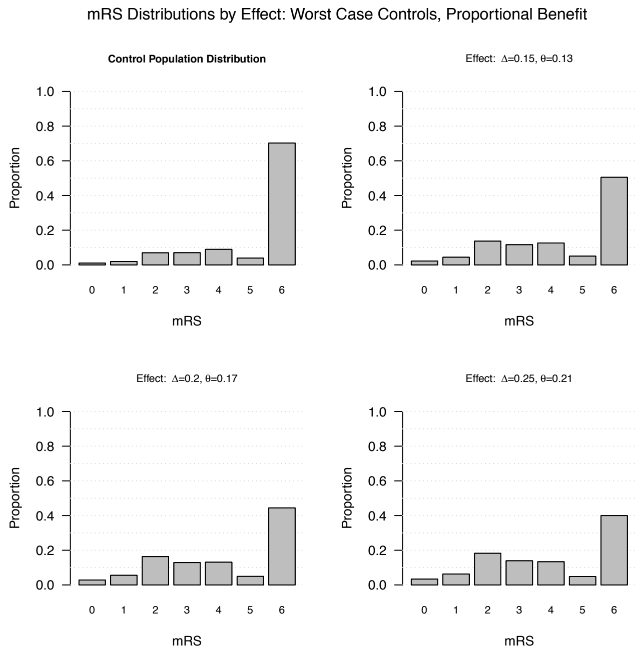

Table 9: mRS Distributions by Effect: Worse Case Controls

	Δ	θ	mRS Proportion 0	1	2	3	4	5	6
Control	0.15	0.13	0.01	0.02	0.07	0.07	0.09	0.04	0.70
Small	0.15	0.13	0.02	0.04	0.14	0.12	0.13	0.05	0.50
Medium	0.20	0.17	0.03	0.05	0.16	0.13	0.13	0.05	0.45
Large	0.25	0.21	0.03	0.06	0.18	0.14	0.13	0.05	0.40

Table 10: Worst Case Controls: ABG/Lobar=(0.60,0.40); Drop=10%; Long=0.4

Parameters Acc	Drop	Long	Effect Size Δ_1	Δ_2	Avg Sample Size Overall	N_1	N_2	Months	Power	StopPredSucc Win	Lose	Stop Futility	StopMax Win	Lose	Successful Indications ABG	Lobar	Both
5	10	0.4	0	0	206	117	89	56.2	0.021	0.009	0.004	0.811	0.012	0.164	0.001	0.005	0.015
			small	small	209	125	84	50.5	0.906	0.825	0.012	0.017	0.081	0.065	0.041	0.029	0.836
			med	med	173	104	69	41.5	0.991	0.978	0.004	0.002	0.013	0.003	0.017	0.005	0.969
			small	0	250	172	78	64.8	0.664	0.491	0.009	0.104	0.173	0.223	0.356	0.000	0.308
			med	small	186	112	73	44.7	0.983	0.950	0.007	0.003	0.033	0.007	0.046	0.008	0.929
			large	small	170	103	67	41.1	0.996	0.990	0.003	0.001	0.006	0.000	0.043	0.003	0.950
			large	0	205	136	69	52.0	0.976	0.929	0.004	0.006	0.047	0.014	0.449	0.000	0.527
			0	small	261	116	146	80.0	0.490	0.323	0.005	0.159	0.167	0.346	0.000	0.322	0.168
			0	med	256	113	143	78.3	0.734	0.575	0.014	0.051	0.159	0.201	0.000	0.485	0.249
15			0	0	224	121	103	24.7	0.018	0.005	0.002	0.806	0.013	0.174	0.004	0.001	0.013
			small	small	255	149	106	24.7	0.897	0.633	0.015	0.021	0.264	0.065	0.047	0.069	0.781
			med	med	217	130	87	21.2	0.988	0.918	0.002	0.005	0.070	0.004	0.011	0.017	0.960
			small	0	274	179	95	27.2	0.524	0.230	0.010	0.161	0.294	0.304	0.283	0.001	0.240
			med	small	232	139	93	22.4	0.968	0.842	0.008	0.007	0.126	0.017	0.045	0.021	0.902
			large	small	213	129	84	21.0	0.993	0.936	0.002	0.002	0.057	0.003	0.044	0.007	0.942
			large	0	255	167	88	25.2	0.938	0.723	0.011	0.011	0.215	0.039	0.470	0.000	0.468
			0	small	278	124	154	32.4	0.410	0.169	0.007	0.165	0.241	0.417	0.002	0.282	0.126
			0	med	280	124	156	32.5	0.690	0.347	0.010	0.054	0.343	0.246	0.000	0.492	0.198
30			0	0	282	158	124	17.0	0.022	0.002	0.000	0.647	0.020	0.329	0.005	0.006	0.011
			small	small	295	175	120	16.5	0.879	0.213	0.000	0.020	0.666	0.096	0.018	0.041	0.820
			med	med	286	171	115	16.1	0.985	0.503	0.000	0.000	0.482	0.013	0.013	0.014	0.958
			small	0	297	180	117	16.8	0.484	0.036	0.002	0.110	0.448	0.395	0.181	0.004	0.299
			med	small	290	174	116	16.2	0.960	0.393	0.002	0.003	0.567	0.032	0.035	0.009	0.916
			large	small	284	170	114	16.0	0.989	0.537	0.000	0.001	0.452	0.007	0.034	0.005	0.950
			large	0	295	181	115	16.6	0.888	0.206	0.001	0.010	0.682	0.099	0.369	0.001	0.518
			0	small	297	160	137	17.9	0.281	0.016	0.000	0.113	0.265	0.603	0.001	0.142	0.138
			0	med	298	161	137	18.0	0.477	0.043	0.001	0.042	0.434	0.480	0.001	0.263	0.213

*Group numbers correspond to 1) Anterior Basal Ganglia; 2) Lobar

5.3 Best Case Control Distribution

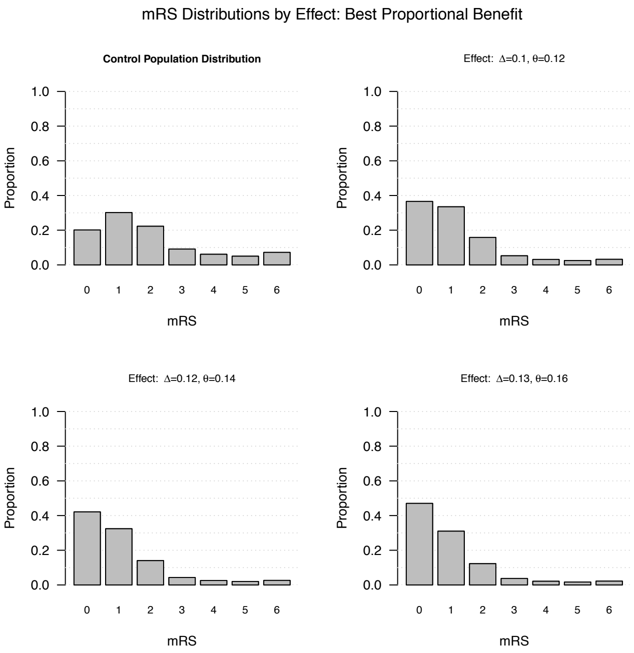

Table 11: mRS Distributions by Effect: Best Case Controls

			mRS Proportion						
	Δ	θ	0	1	2	3	4	5	6
Control	0.10	0.12	0.20	0.30	0.22	0.09	0.06	0.05	0.07
Small	0.10	0.12	0.37	0.33	0.16	0.05	0.03	0.02	0.03
Medium	0.12	0.14	0.42	0.32	0.14	0.04	0.03	0.02	0.03
Large	0.13	0.16	0.47	0.31	0.12	0.04	0.02	0.02	0.02

Table 12: Best Case Controls: ABG/Lobar=(0.60,0.40); Drop=10%; Long=0.4

Parameters			Effect Size		Avg Sample Size					StopPredSucc		Stop	StopMax		Successful Indications		
Acc	Drop	Long	Δ_1	Δ_2	Overall	N_1	N_2	Months	Power	Win	Lose	Futility	Win	Lose	ABG	Lobar	Both
5	10	0.4	0	0	192	109	82	51.8	0.019	0.009	0.004	0.893	0.010	0.084	0.001	0.003	0.015
			small	small	197	117	79	48.3	0.945	0.883	0.010	0.018	0.062	0.027	0.059	0.046	0.840
			med	med	175	105	70	42.4	0.992	0.983	0.001	0.003	0.009	0.004	0.033	0.016	0.943
			small	0	238	166	72	62.7	0.730	0.580	0.010	0.111	0.150	0.149	0.445	0.000	0.285
			med	small	183	111	73	44.5	0.976	0.956	0.007	0.010	0.020	0.007	0.060	0.015	0.901
			large	small	174	105	68	42.2	0.994	0.981	0.002	0.004	0.013	0.000	0.057	0.008	0.929
			large	0	214	145	68	55.3	0.952	0.877	0.008	0.021	0.075	0.019	0.531	0.001	0.420
			0	small	255	107	147	80.7	0.583	0.420	0.005	0.167	0.163	0.245	0.000	0.437	0.146
			0	med	251	106	144	79.2	0.754	0.603	0.011	0.079	0.151	0.156	0.000	0.564	0.190
15			0	0	213	115	98	23.9	0.021	0.004	0.002	0.864	0.017	0.112	0.005	0.004	0.012
			small	small	243	142	101	24.0	0.935	0.764	0.007	0.025	0.171	0.031	0.059	0.091	0.785
			med	med	220	130	89	21.7	0.987	0.917	0.003	0.006	0.070	0.004	0.032	0.051	0.904
			small	0	268	178	91	27.2	0.593	0.308	0.012	0.178	0.285	0.216	0.397	0.005	0.191
			med	small	230	137	93	22.5	0.971	0.867	0.006	0.010	0.104	0.013	0.061	0.049	0.861
			large	small	219	132	87	21.5	0.985	0.924	0.003	0.008	0.061	0.004	0.061	0.023	0.901
			large	0	259	173	86	26.1	0.874	0.642	0.013	0.052	0.232	0.059	0.548	0.002	0.324
			0	small	274	117	157	32.9	0.512	0.254	0.006	0.170	0.258	0.310	0.002	0.405	0.105
			0	med	276	117	159	33.1	0.702	0.382	0.009	0.085	0.320	0.204	0.000	0.564	0.138
30			0	0	278	154	123	17.0	0.021	0.002	0.000	0.756	0.019	0.220	0.004	0.011	0.006
			small	small	293	174	119	16.5	0.918	0.294	0.001	0.016	0.624	0.051	0.032	0.065	0.821
			med	med	288	171	116	16.2	0.976	0.477	0.001	0.000	0.499	0.014	0.021	0.030	0.925
			small	0	296	180	116	16.8	0.556	0.049	0.001	0.129	0.507	0.300	0.278	0.007	0.271
			med	small	290	173	117	16.3	0.957	0.410	0.002	0.003	0.547	0.027	0.042	0.027	0.888
			large	small	287	172	115	16.2	0.981	0.491	0.000	0.001	0.490	0.013	0.047	0.013	0.921
			large	0	296	182	114	16.7	0.817	0.149	0.002	0.033	0.668	0.141	0.433	0.001	0.383
			0	small	296	156	139	18.2	0.338	0.026	0.000	0.131	0.312	0.525	0.002	0.219	0.117
			0	med	297	157	141	18.2	0.490	0.053	0.000	0.068	0.437	0.440	0.001	0.337	0.152

* Group numbers correspond to 1) Anterior Basal Ganglia; 2) Lobar

5.4 Expected Control Distribution, Localized Benefit 1-3

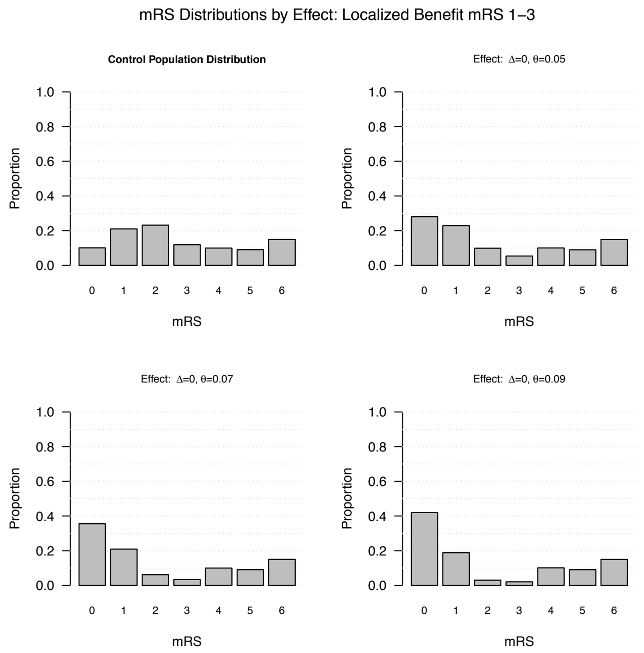

Table 13: mRS Distributions by Effect: Expected Controls, Localized Benefit 1-3

	Δ	θ	mRS Proportion 0	1	2	3	4	5	6
Control	0.00	0.05	0.10	0.21	0.23	0.12	0.10	0.09	0.15
Small	0.00	0.05	0.28	0.23	0.10	0.05	0.10	0.09	0.15
Medium	0.00	0.07	0.35	0.21	0.06	0.03	0.10	0.09	0.15
Large	0.00	0.09	0.42	0.19	0.03	0.02	0.10	0.09	0.15

Table 14: Localized Benefit 1-3: ABG/Lobar=(0.60,0.40); Drop=10%; Long=0.4

Parameters Acc	Drop	Long	Effect Size Δ_1	Δ_2	Avg Sample Size Overall	N_1	N_2	Months	Power	StopPredSucc Win	Lose	Stop Futility	StopMax Win	Lose	Successful Indications ABG	Lobar	Both
5	10	0.4	0	0	220	126	94	59.5	0.026	0.010	0.005	0.701	0.016	0.268	0.001	0.005	0.020
			small	small	257	149	108	66.9	0.232	0.126	0.007	0.308	0.106	0.453	0.018	0.012	0.202
			med	med	259	152	107	65.9	0.352	0.226	0.007	0.204	0.126	0.437	0.024	0.016	0.312
			small	0	246	155	91	64.2	0.116	0.061	0.005	0.465	0.055	0.414	0.023	0.000	0.093
			med	small	259	155	104	65.7	0.308	0.190	0.005	0.241	0.118	0.446	0.031	0.010	0.267
			large	small	258	158	101	64.9	0.369	0.237	0.009	0.202	0.132	0.420	0.048	0.008	0.313
			large	0	258	169	89	66.2	0.238	0.130	0.008	0.297	0.108	0.457	0.065	0.000	0.173
			0	small	242	127	116	67.5	0.069	0.039	0.006	0.495	0.030	0.430	0.000	0.019	0.050
			0	med	250	126	124	71.1	0.115	0.063	0.006	0.414	0.052	0.465	0.000	0.036	0.079
15			0	0	235	128	107	25.6	0.019	0.004	0.003	0.705	0.015	0.273	0.003	0.000	0.016
			small	small	268	148	120	27.8	0.171	0.049	0.006	0.344	0.122	0.479	0.018	0.019	0.134
			med	med	274	153	122	27.9	0.292	0.102	0.008	0.237	0.190	0.463	0.023	0.034	0.235
			small	0	255	150	105	26.4	0.085	0.024	0.002	0.506	0.061	0.407	0.021	0.002	0.062
			med	small	271	154	118	27.6	0.225	0.080	0.009	0.284	0.145	0.481	0.025	0.023	0.177
			large	small	273	158	115	27.3	0.292	0.105	0.007	0.244	0.187	0.457	0.037	0.019	0.236
			large	0	266	162	104	26.9	0.167	0.047	0.005	0.356	0.120	0.472	0.050	0.001	0.116
			0	small	257	129	128	28.5	0.060	0.013	0.001	0.490	0.047	0.449	0.003	0.015	0.042
			0	med	263	129	134	29.3	0.090	0.023	0.006	0.419	0.067	0.485	0.002	0.030	0.058
30			0	0	285	161	124	17.0	0.020	0.001	0.000	0.540	0.019	0.440	0.004	0.001	0.015
			small	small	294	170	124	17.0	0.169	0.005	0.000	0.220	0.164	0.607	0.009	0.013	0.147
			med	med	296	173	123	16.9	0.300	0.013	0.001	0.145	0.287	0.552	0.019	0.019	0.262
			small	0	291	170	121	16.9	0.086	0.002	0.000	0.340	0.084	0.572	0.014	0.002	0.070
			med	small	295	173	122	16.9	0.237	0.007	0.000	0.187	0.230	0.572	0.019	0.012	0.206
			large	small	296	174	122	16.8	0.307	0.014	0.001	0.140	0.293	0.549	0.026	0.015	0.266
			large	0	294	175	119	16.8	0.161	0.005	0.000	0.228	0.156	0.608	0.035	0.004	0.122
			0	small	290	162	128	17.3	0.060	0.003	0.000	0.372	0.057	0.567	0.002	0.009	0.049
			0	med	292	162	130	17.4	0.079	0.003	0.000	0.294	0.076	0.626	0.001	0.016	0.062

*Group numbers correspond to 1) Anterior Basal Ganglia; 2) Lobar

5.5 Expected Control Distribution, Localized Benefit 4-6

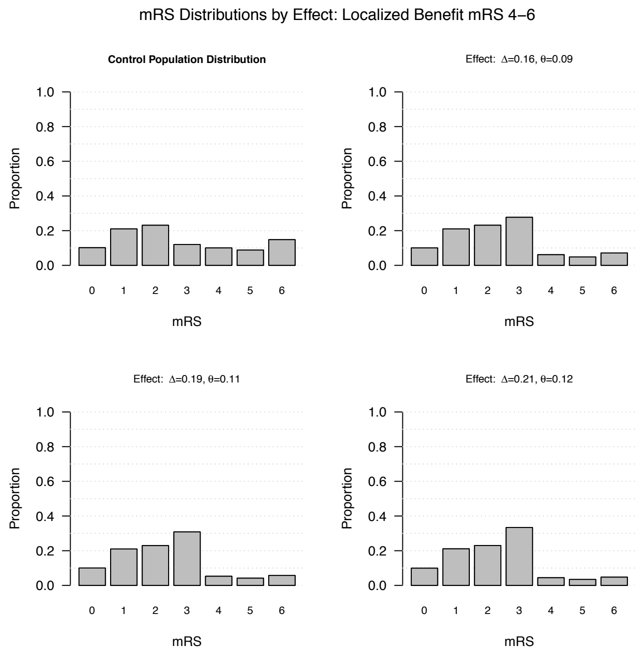

Table 15: mRS Distributions by Effect: Expected Controls, Localized Benefit 4-6

	Δ	θ	mRS Proportion 0	1	2	3	4	5	6
Control	0.16	0.09	0.10	0.21	0.23	0.12	0.10	0.09	0.15
Small	0.16	0.09	0.10	0.21	0.23	0.28	0.06	0.05	0.07
Medium	0.19	0.11	0.10	0.21	0.23	0.31	0.05	0.04	0.06
Large	0.21	0.12	0.10	0.21	0.23	0.33	0.05	0.03	0.05

Table 16: Localized Benefit 4-6: ABG/Lobar=(0.60,0.40); Drop=10%; Long=0.4

Parameters Acc	Drop	Long	Effect Size Δ_1	Δ_2	Avg Sample Size Overall	N_1	N_2	Months	Power	StopPredSucc Win	Lose	Stop Futility	StopMax Win	Lose	Successful Indications ABG	Lobar	Both
5	10	0.4	0	0	206	117	89	56.2	0.021	0.009	0.004	0.811	0.012	0.164	0.001	0.005	0.015
			small	small	242	142	100	61.0	0.638	0.508	0.012	0.109	0.130	0.241	0.051	0.047	0.540
			med	med	229	135	93	56.3	0.795	0.671	0.013	0.053	0.124	0.139	0.052	0.042	0.701
			small	0	252	170	82	65.7	0.393	0.247	0.006	0.281	0.146	0.320	0.192	0.001	0.200
			med	small	234	141	93	58.0	0.737	0.610	0.013	0.074	0.127	0.176	0.076	0.029	0.632
			large	small	228	139	89	56.0	0.798	0.682	0.013	0.048	0.116	0.141	0.088	0.017	0.693
			large	0	252	173	79	65.3	0.599	0.440	0.008	0.128	0.159	0.265	0.316	0.000	0.283
			0	small	255	118	138	77.2	0.246	0.141	0.011	0.332	0.105	0.411	0.000	0.144	0.102
			0	med	260	117	143	79.0	0.348	0.210	0.012	0.251	0.138	0.389	0.000	0.214	0.134
15			0	0	224	121	103	24.7	0.018	0.005	0.002	0.806	0.013	0.174	0.004	0.001	0.013
			small	small	271	153	118	27.2	0.558	0.293	0.014	0.130	0.265	0.295	0.047	0.070	0.441
			med	med	267	154	113	26.2	0.755	0.451	0.015	0.065	0.304	0.161	0.063	0.084	0.608
			small	0	263	165	98	26.8	0.263	0.098	0.006	0.361	0.165	0.369	0.125	0.002	0.136
			med	small	269	157	112	26.5	0.670	0.376	0.020	0.088	0.294	0.215	0.071	0.057	0.542
			large	small	267	158	109	26.0	0.753	0.444	0.017	0.064	0.309	0.164	0.097	0.040	0.616
			large	0	272	176	96	27.2	0.463	0.188	0.012	0.195	0.275	0.328	0.249	0.003	0.211
			0	small	268	123	145	31.0	0.209	0.072	0.005	0.334	0.137	0.452	0.002	0.125	0.082
			0	med	274	124	150	31.8	0.286	0.102	0.003	0.249	0.184	0.462	0.003	0.184	0.099
30			0	0	282	158	124	17.0	0.022	0.002	0.000	0.647	0.020	0.329	0.005	0.006	0.011
			small	small	297	174	122	16.8	0.620	0.069	0.001	0.076	0.551	0.297	0.039	0.066	0.515
			med	med	296	175	121	16.7	0.747	0.113	0.003	0.038	0.634	0.206	0.026	0.055	0.666
			small	0	294	176	118	16.8	0.253	0.007	0.000	0.235	0.246	0.507	0.085	0.008	0.160
			med	small	297	175	121	16.7	0.691	0.086	0.003	0.058	0.605	0.243	0.041	0.040	0.610
			large	small	296	176	120	16.6	0.754	0.115	0.003	0.043	0.639	0.193	0.046	0.037	0.671
			large	0	296	179	117	16.8	0.443	0.031	0.001	0.135	0.412	0.416	0.161	0.005	0.277
			0	small	293	159	134	17.8	0.150	0.008	0.000	0.246	0.142	0.600	0.001	0.066	0.083
			0	med	295	159	136	17.9	0.206	0.011	0.000	0.175	0.195	0.613	0.001	0.099	0.106

*Group numbers correspond to 1) Anterior Basal Ganglia; 2) Lobar

5.6 Simulation Summary

We make the following general observations based on the simulation results:

- Slower accrual generally has better performance
- Power is relatively robust to more extreme control mRS distributions
- Dropout decreases power
- Prevalence of locations has differential impact depending on which location has a treatment effect
- Longitudinal model with correlation equal to 0.4 leads to small power increase
- Localized treatment effect (mRS 1-3 or 4-6 only) can cause low power because difficult to obtain large observed difference in means

5.7 Computational Details

The simulation report reflects the design parameters contained within the `MinSubAdaptive2min300Med_25_v4.facts` file. For each scenario, 1000 trials were simulated on 05/12/2016 16:19:00 using FACTS version 4.0.10 with random seeds 3500 and 1. Posterior quantities were calculated using MCMC samples from a single chain of length 2500 after a burn-in of 1000 samples. The R software package was used to summarize the simulation output and to create graphics and tables for this report. This document was typeset with $\LaTeX\,2_\varepsilon$.

6 Example Trials

Example trials are shown to illustrate the adaptive algorithm. Results are presented for each trial update for 4 distinct trials. Each table summarizes the following information:

- Update: Current trial update
- Month: Length of study in months at time of update
- Number Patients: Number of patients enrolled (Enr) and with complete data (Compl)
- Treatment Benefit: Observed mean treatment benefit (treatment minus control) with respect to utility-weighted mRS for 1) ABG location, 2) Lobar location, and 3) All patients with corresponding standard error
- Posterior Prob Fut(ABG): Posterior probability of futility in ABG location
- Posterior Prob Fut(Lobar): Posterior probability of futility in Lobar location
- Posterior Prob Succ(All): Posterior probability of success (superiority) in all enrolled patients
- Pval: P-value for surgery vs. control, all enrolled patients
- Stopping Indicators Fut(ABG): Whether the trial meets futility criteria for ABG location
- Stopping Indicators Fut(Lobar): Whether the trial meets futility criteria for Lobar location
- Stopping Indicators Succ(All): Whether the trial meets predicted success criteria for all enrolled patients.

The following graphs are also shown for each trial update: 1) a histogram of mRS distributions by location and treatment; and 2) a line plot showing the number of patients enrolled by location (where "X" indicates accrual stop).

Example trials are generated from the following scenario: Maximum accrual of 15 patients/mth, proportional treatment effect, expected mRS control distribution, no dropouts, prevalence of (ABG,Lobar) equal to (0.6,0.4), and a linear correlation between 90 and 180-day outcomes of 0.4.

6.1 Example 1

In Example 1, trial update 1 occurs 14 months into the study. There are 150 patients enrolled, but only 51 have complete 180-day outcomes. The mean treatment benefit is 0.04, -0.01, and 0.02 for ABG, Lobar, and both locations combined, respectively. Neither location meets futility criteria (Fut(location)$\leq$ 0.20 and at least 30 patients complete 180 data within a location). Also, the trial does not meet meet predicted success criteria (Succ(All)$\geq$ 0.99 and at least 60 patients with complete 180 day data). Hence the trial continues. At the second update, with 175 patients enrolled and 85 with complete 180-day outcome data, the mean benefit is -0.07 and -0.06 for ABG and Lobar, respectively. The probability of futility is 0.064 and 0.084 for ABG and Lobar, respectively, which are both less than the 0.20 to trigger early stopping (and at least 30 patients in each location have complete 180-day outcomes). Hence the trial stops for futility. Trial update 3 shows the final results after all 175 patients have observed 180-day outcomes.

6.1.1 Example 1: Update 1

Table 17: Example 1: Update 1

Update	Month	Number Patients Enr	Compl	Treatment Benefit ABG	Lobar	All(SE)	Posterior Prob Fut(ABG)	Fut(Lb)	Succ(All)	Pval	Stopping Indicators Fut(ABG)	Fut(Lb)	Succ(All)
1	14	150	51	0.04	-0.01	0.02 (0.1)	0.369	0.246	0.572	0.428	no	no	no

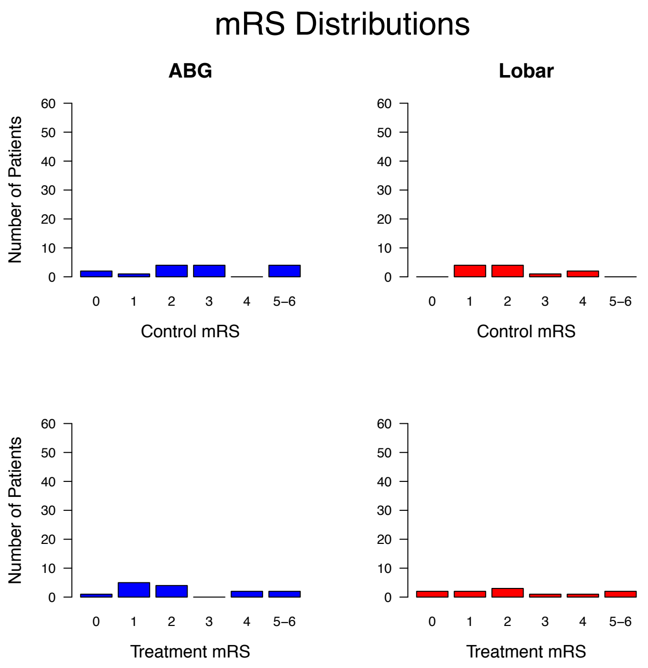

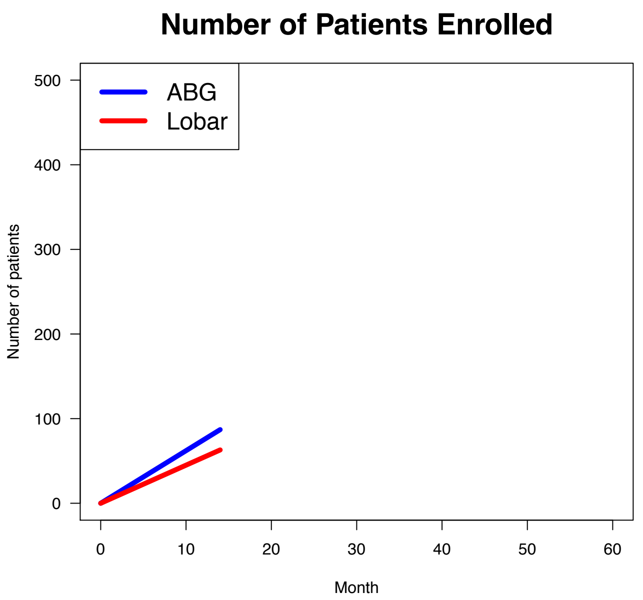

6.1.2 Example 1: Update 2

Table 18: Example 1: Update 2

Update	Month	Number Patients Enr	Compl	Treatment Benefit ABG	Lobar	All(SE)	Posterior Prob Fut(ABG)	Fut(Lb)	Succ(All)	Pval	Stopping Indicators Fut(ABG)	Fut(Lb)	Succ(All)
2	16	175	85	-0.07	-0.06	-0.06 (0.07)	0.064	0.084	0.233	0.791	yes	yes	no

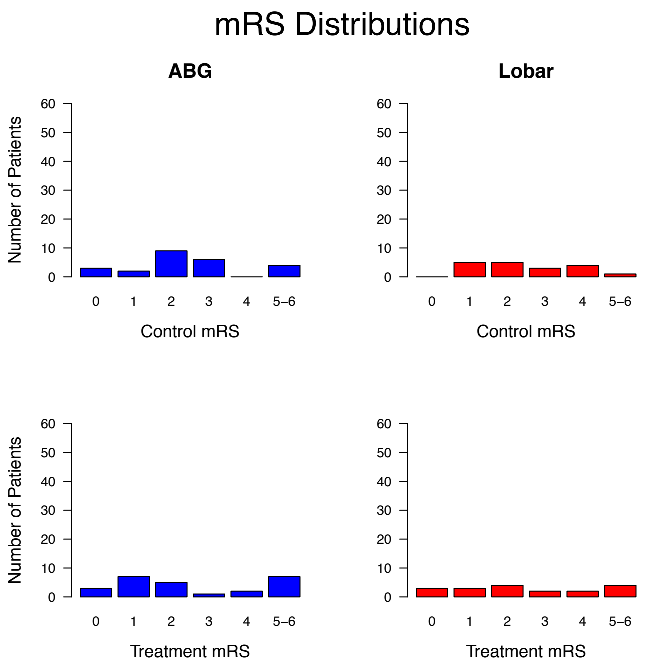

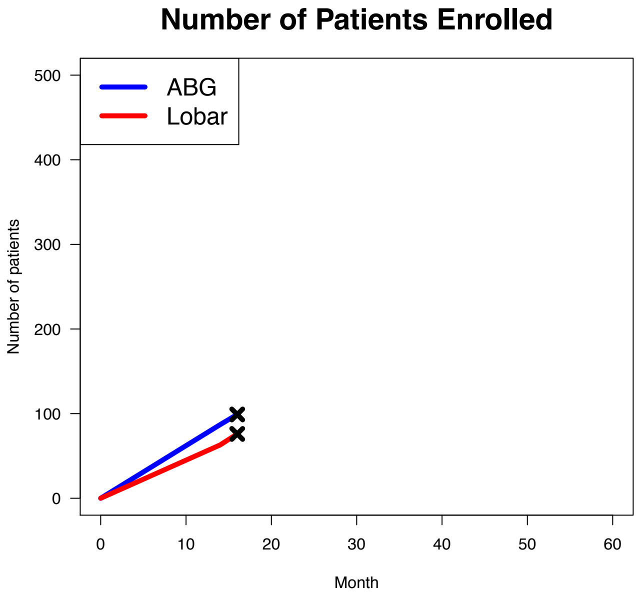

6.1.3 Example 1: Final

Table 19: Example 1: Final

Update	Month	Number Patients Enr	Number Patients Compl	Treatment Benefit ABG	Treatment Benefit Lobar	Treatment Benefit All(SE)	Posterior Prob Fut(ABG)	Posterior Prob Fut(Lb)	Posterior Prob Succ(All)	Posterior Prob Pval	Stopping Indicators Fut(ABG)	Stopping Indicators Fut(Lb)	Stopping Indicators Succ(All)
Final	22	175	175	0.00	0.05	0.02 (0.05)	0.115	0.353	0.664	0.334	yes	yes	no

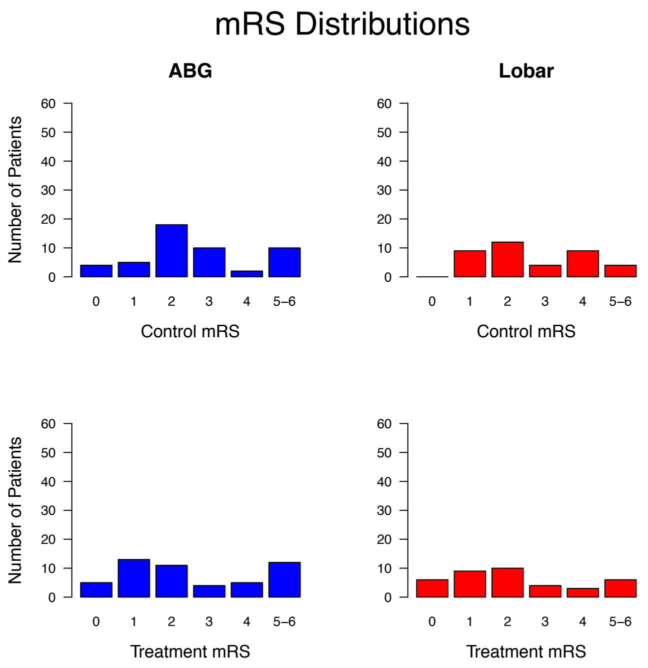

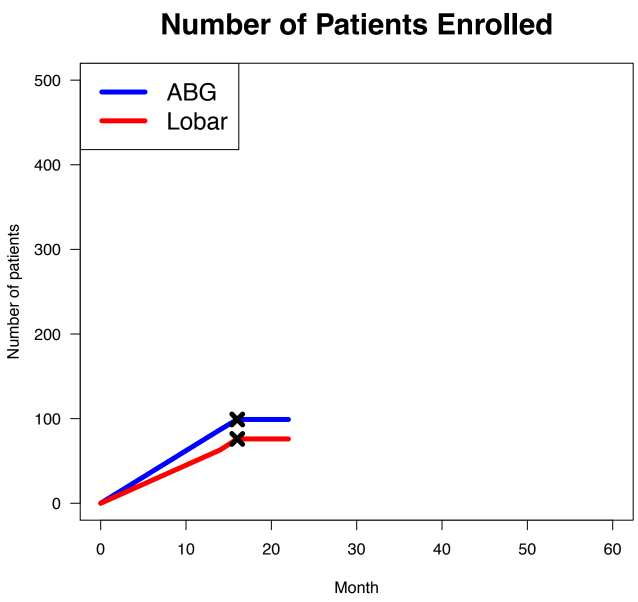

6.2 Example 2

Example 2 is a case in which the predicted success criteria (≥ 0.99) is met at the first trial update, with posterior prob Succ(All) = 0.992, with 68 patients that have complete 180-day outcomes. Hence the trial stops accrual for predicted success. After 180 days, all 150 enrolled patients have complete data, at which point the decisive analysis is performed and the trial meets superiority criteria (Post Prob Succ ≥ 0.975). Hence the trial demonstrates superiority of surgery versus control for both ABG and Lobar locations with a mean benefit of 0.16.

6.2.1 Example 2: Update 1

Table 20: Example 2: Update 1

Update	Month	Number Patients Enr	Compl	Treatment Benefit ABG	Lobar	All(SE)	Posterior Prob Fut(ABG)	Fut(Lb)	Succ(All)	Pval	Stopping Indicators Fut(ABG)	Fut(Lb)	Succ(All)
1	15	150	68	0.22	0.18	0.2 (0.08)	0.951	0.856	0.992	0.006	no	no	yes

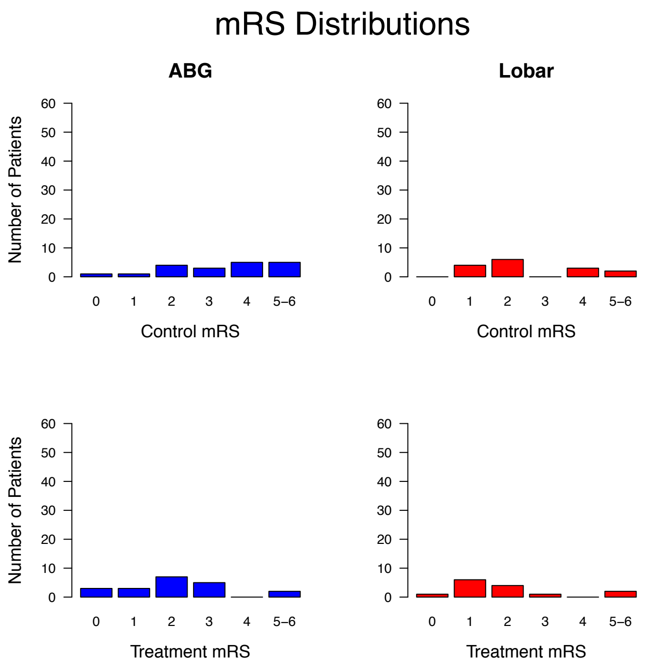

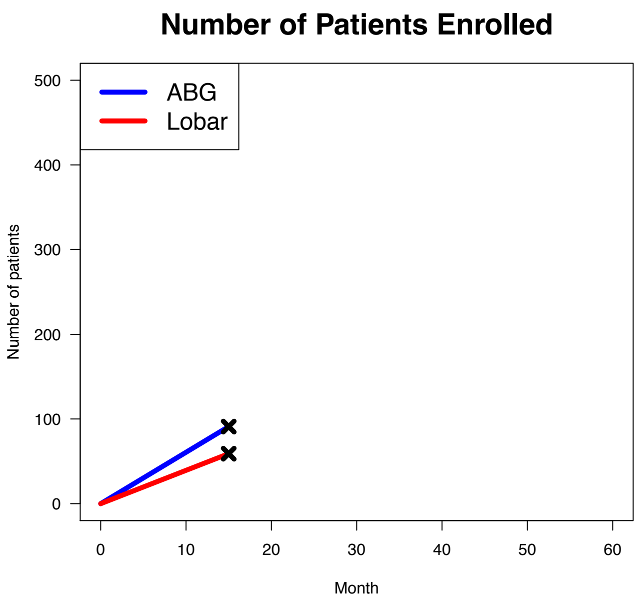

6.2.2 Example 2: Final

Table 21: Example 2: Final

Update	Month	Number Patients Enr	Number Patients Compl	Treatment Benefit ABG	Treatment Benefit Lobar	Treatment Benefit All(SE)	Posterior Prob Fut(ABG)	Posterior Prob Fut(Lb)	Posterior Prob Succ(All)	Pval	Stopping Indicators Fut(ABG)	Stopping Indicators Fut(Lb)	Stopping Indicators Succ(All)
Final	21	150	150	0.18	0.15	0.16 (0.06)	0.946	0.845	0.998	0.002	no	no	yes

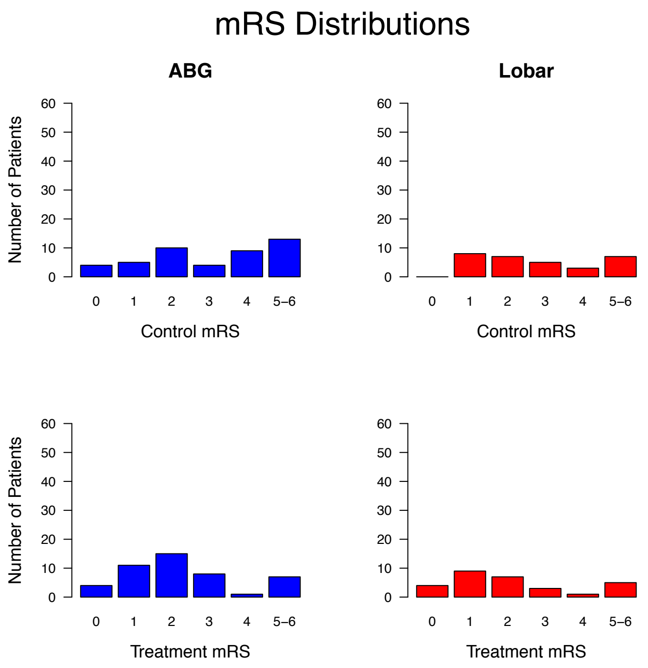

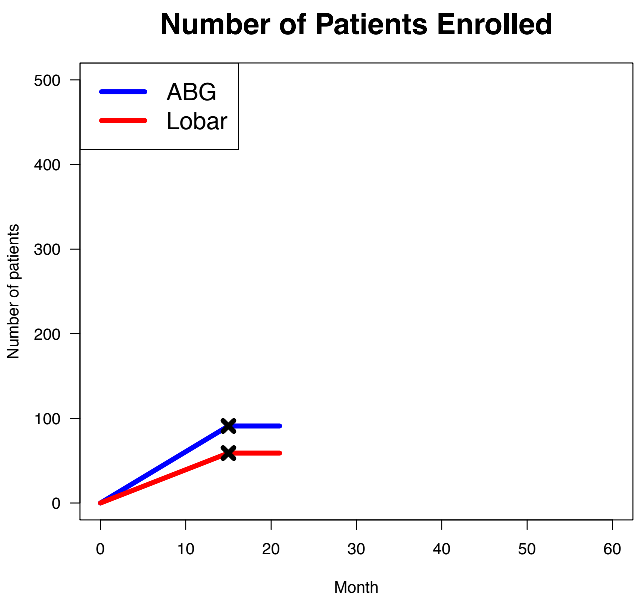

6.3 Example 3

In example 3, ABG meets futility criteria at the first trial update; hence accrual stops within ABG but continues for the Lobar location. At trial update 5 with 250 patients enrolled (215 with complete outcome data), the trial meets predicted success criteria and accrual is stopped in the remaining Lobar location. After waiting an additional 180 days, a decisive analysis is performed which meets final evaluation criteria for superiority with Post Prob Succ = 0.990 and mean treatment benefit of 0.10. Because ABG was dropped (enrichment), superiority of the surgery versus control is only claimed in the Lobar location.

6.3.1 Example 3: Update 1

Table 22: Example 3: Update 1

Update	Month	Number Patients Enr	Compl	Treatment Benefit ABG	Lobar	All(SE)	Posterior Prob Fut(ABG)	Fut(Lb)	Succ(All)	Pval	Stopping Indicators Fut(ABG)	Fut(Lb)	Succ(All)
1	14	150	69	-0.08	0.06	-0.02 (0.1)	0.086	0.455	0.396	0.623	yes	no	no

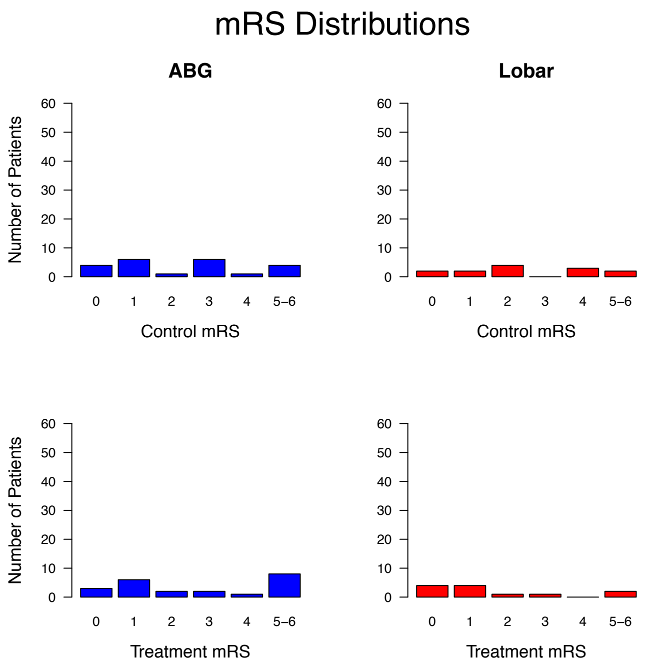

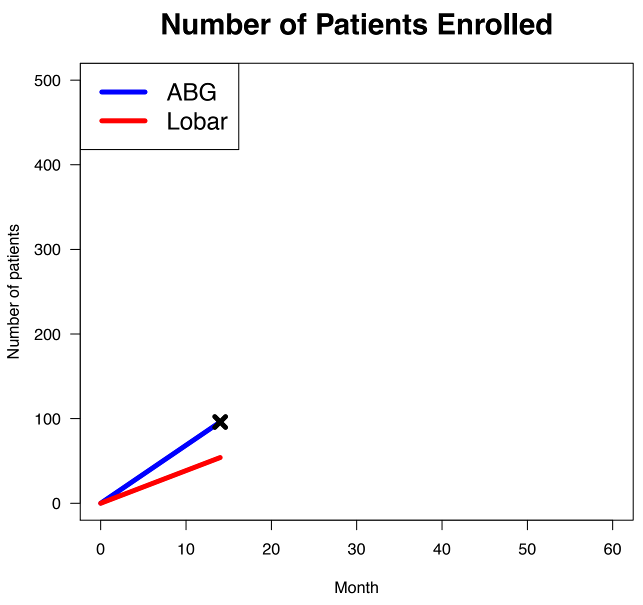

6.3.2 Example 3: Update 2

Table 23: Example 3: Update 2

Update	Month	Number Patients		Treatment Benefit			Posterior Prob				Stopping Indicators		
		Enr	Compl	ABG	Lobar	All(SE)	Fut(ABG)	Fut(Lb)	Succ(All)	Pval	Fut(ABG)	Fut(Lb)	Succ(All)
2	18	175	114	0.02	0.15	0.07 (0.07)	0.232	0.764	0.838	0.176	yes	no	no

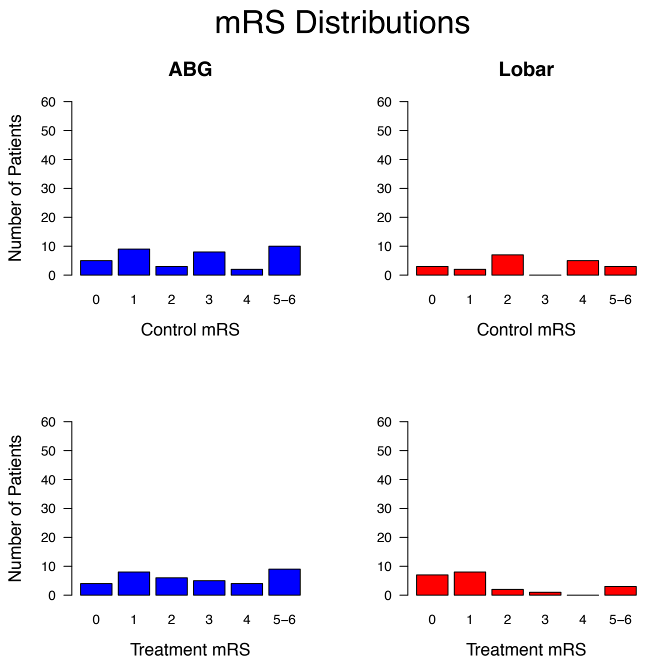

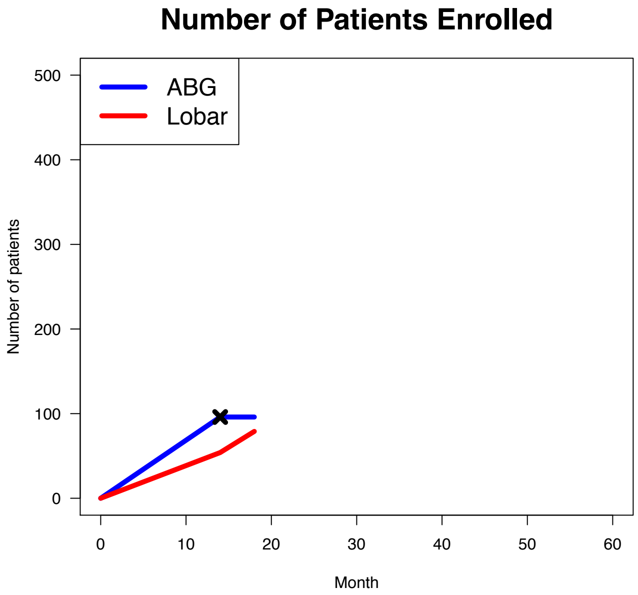

6.3.3 Example 3: Update 3

Table 24: Example 3: Update 3

Update	Month	Number Patients Enr	Compl	Treatment Benefit ABG	Lobar	All(SE)	Posterior Prob Fut(ABG)	Fut(Lb)	Succ(All)	Pval	Stopping Indicators Fut(ABG)	Fut(Lb)	Succ(All)
3	22	200	167	0.03	0.11	0.07 (0.06)	0.285	0.680	0.891	0.110	yes	no	no

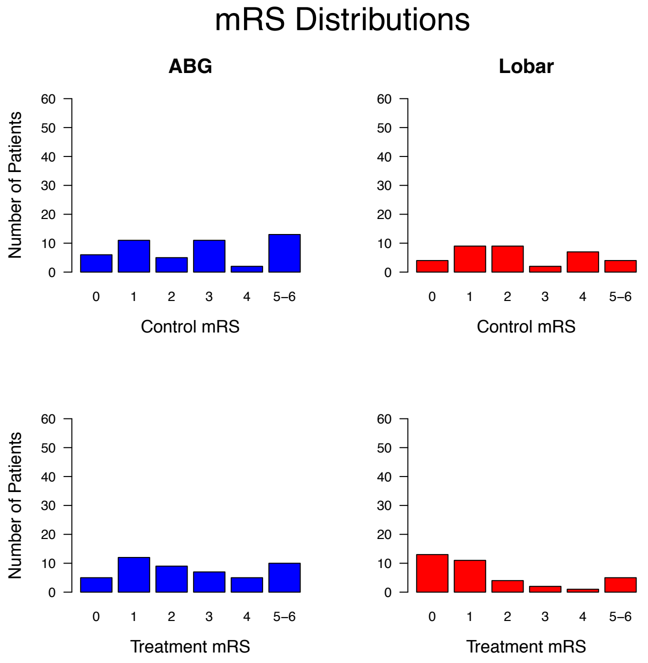

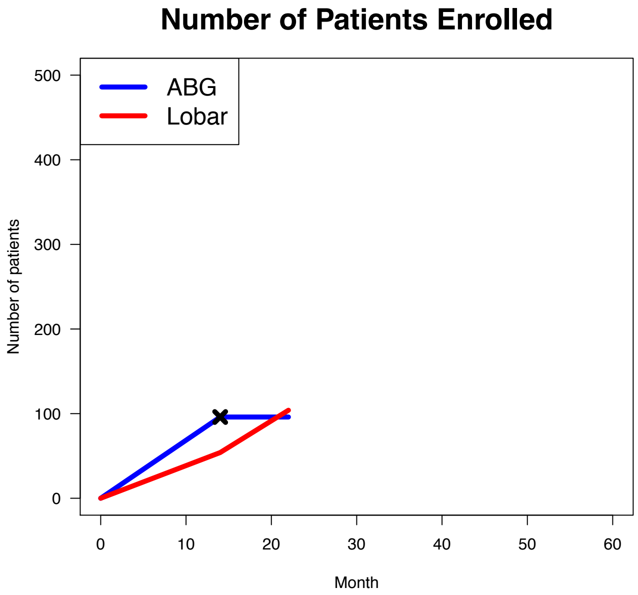

6.3.4 Example 3: Update 4

Table 25: Example 3: Update 4

		Number Patients		Treatment Benefit			Posterior Prob				Stopping Indicators		
Update	Month	Enr	Compl	ABG	Lobar	All(SE)	Fut(ABG)	Fut(Lb)	Succ(All)	Pval	Fut(ABG)	Fut(Lb)	Succ(All)
4	26	225	182	0.04	0.11	0.07 (0.05)	0.272	0.670	0.921	0.093	yes	no	no

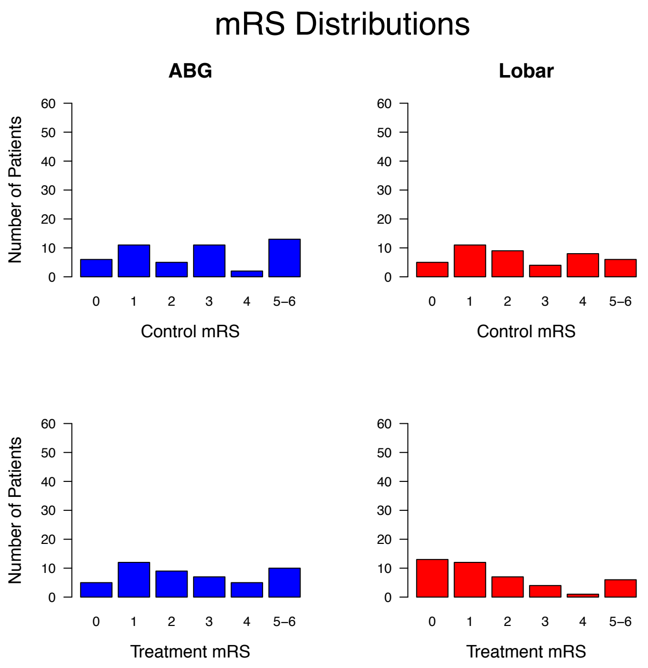

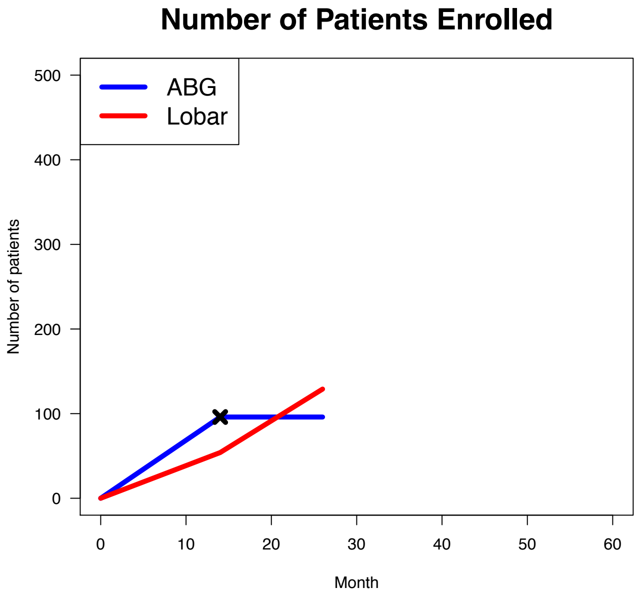

6.3.5 Example 3: Update 5

Table 26: Example 3: Update 5

Update	Month	Number Patients Enr	Compl	Treatment Benefit ABG	Lobar	All(SE)	Posterior Prob Fut(ABG)	Fut(Lb)	Succ(All)	Pval	Stopping Indicators Fut(ABG)	Fut(Lb)	Succ(All)
5	31	250	215	0.05	0.17	0.12 (0.05)	0.374	0.954	0.997	0.008	yes	no	yes

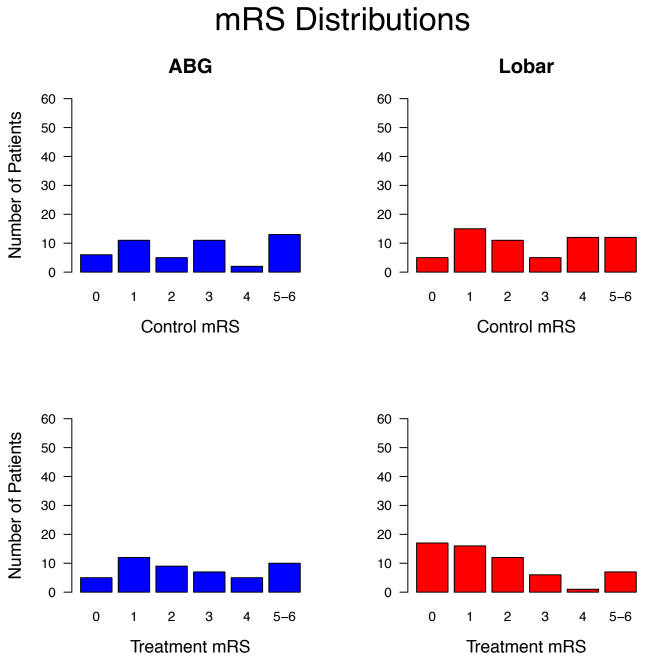

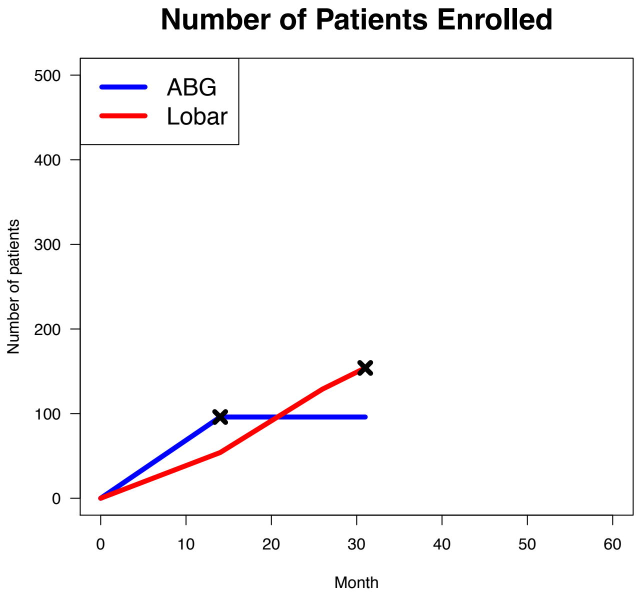

6.3.6 Example 3: Final

Table 27: Example 3: Final

Update	Month	Number Patients Enr	Compl	Treatment Benefit ABG	Lobar	All(SE)	Posterior Prob Fut(ABG)	Fut(Lb)	Succ(All)	Pval	Stopping Indicators Fut(ABG)	Fut(Lb)	Succ(All)
Final	37	250	250	0.05	0.13	0.1 (0.04)	0.346	0.860	0.990	0.013	no	no	yes

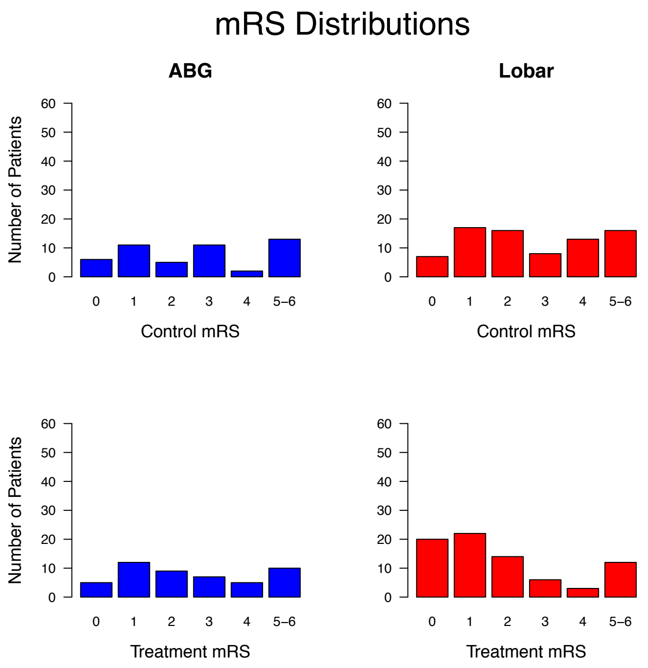

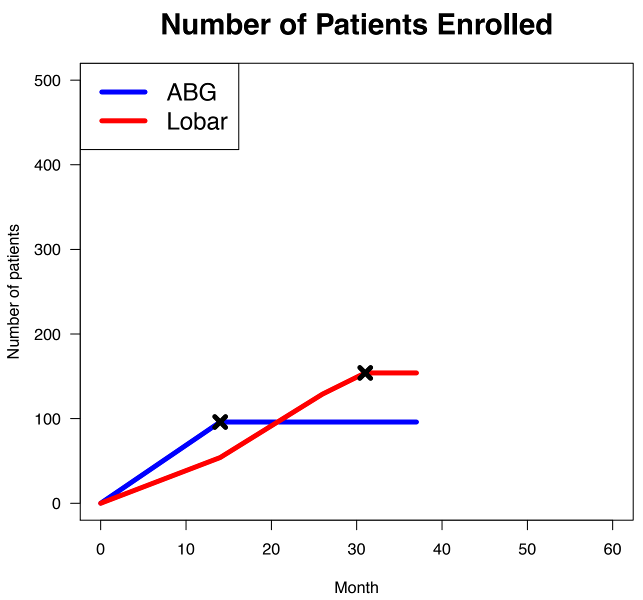

6.4 Example 4

In example 4, none of the early stopping criteria are met in any of the trial updates. The trial enrolls to the maximum sample size (N=300), at which point accrual stops and we wait an additional 180 days to perform the decisive analysis. At 30 months and 300 patients with complete data, the mean treatment benefit is 0.11 and the final evaluation criteria (≥ 0.975) is met for superiority with Post Prob Succ=0.996. Superiority is claimed for both ABG and Lobar locations because neither stopped accrual for futility.

6.4.1 Example 4: Update 1

Table 28: Example 4: Update 1

Update	Month	Number Patients Enr	Compl	Treatment Benefit ABG	Lobar	All(SE)	Posterior Prob Fut(ABG)	Fut(Lb)	Succ(All)	Pval	Stopping Indicators Fut(ABG)	Fut(Lb)	Succ(All)
1	15	150	78	0.11	0.17	0.13 (0.08)	0.666	0.802	0.956	0.056	no	no	no

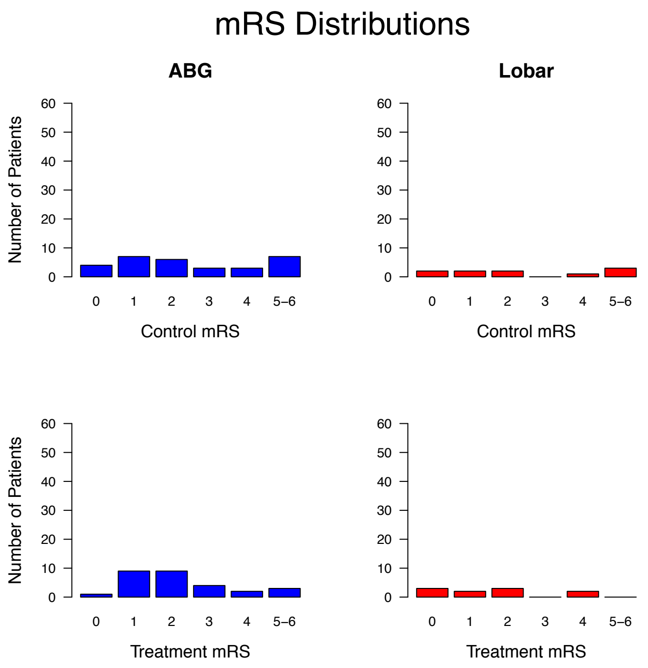

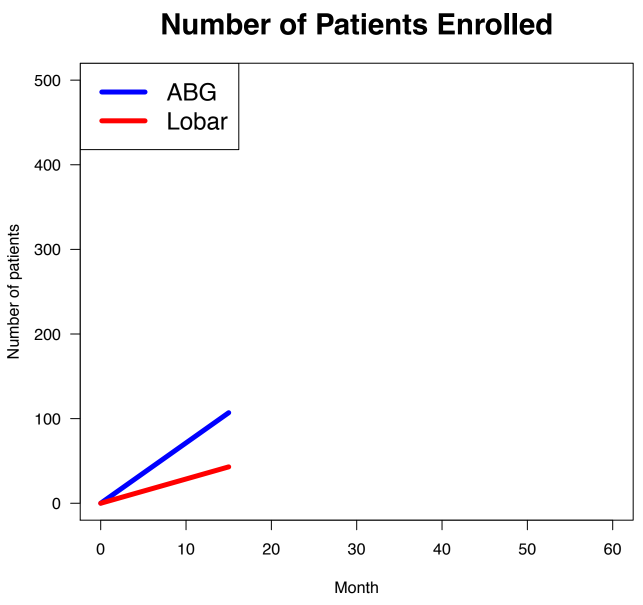

6.4.2 Example 4: Update 2

Table 29: Example 4: Update 2

Update	Month	Number Patients Enr	Compl	Treatment Benefit ABG	Lobar	All(SE)	Posterior Prob Fut(ABG)	Fut(Lb)	Succ(All)	Pval	Stopping Indicators Fut(ABG)	Fut(Lb)	Succ(All)
2	16	175	89	0.09	0.16	0.1 (0.08)	0.554	0.757	0.911	0.093	no	no	no

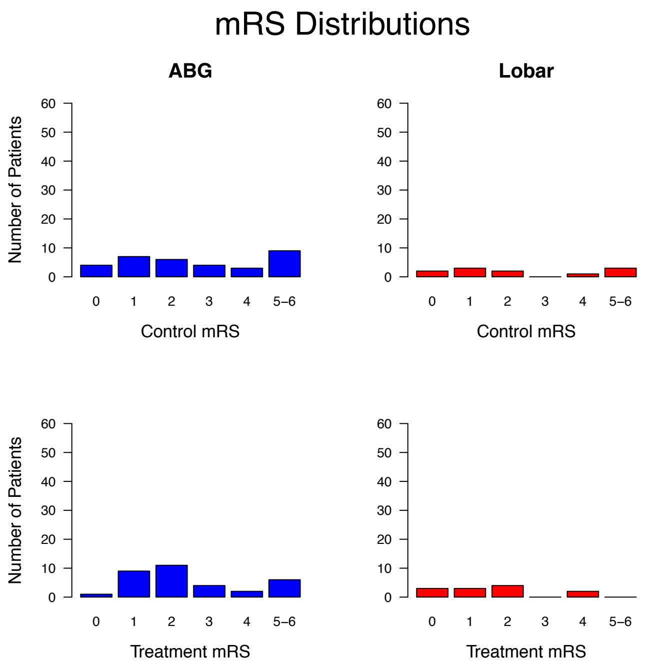

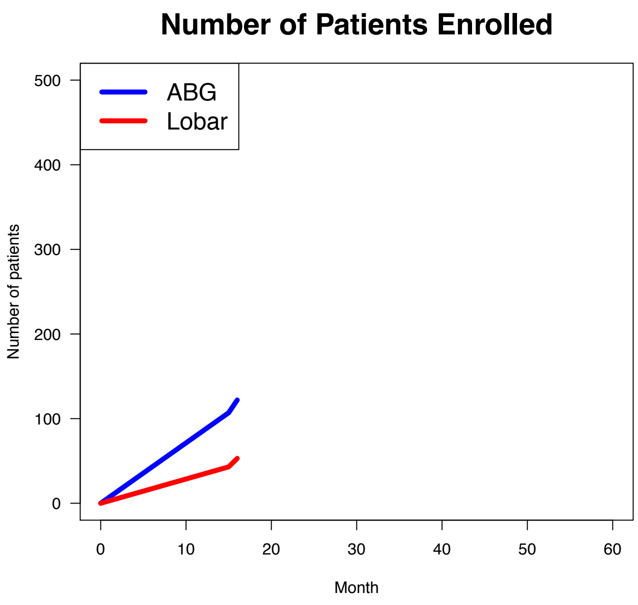

6.4.3 Example 4: Update 3

Table 30: Example 4: Update 3

Update	Month	Number Patients Enr	Compl	Treatment Benefit ABG	Lobar	All(SE)	Posterior Prob Fut(ABG)	Fut(Lb)	Succ(All)	Pval	Stopping Indicators Fut(ABG)	Fut(Lb)	Succ(All)
3	18	200	108	0.08	0.13	0.1 (0.07)	0.544	0.724	0.934	0.075	no	no	no

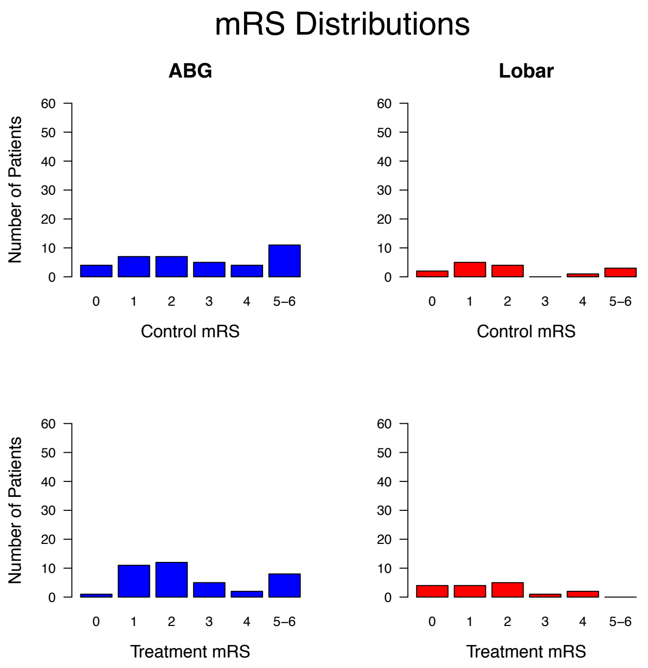

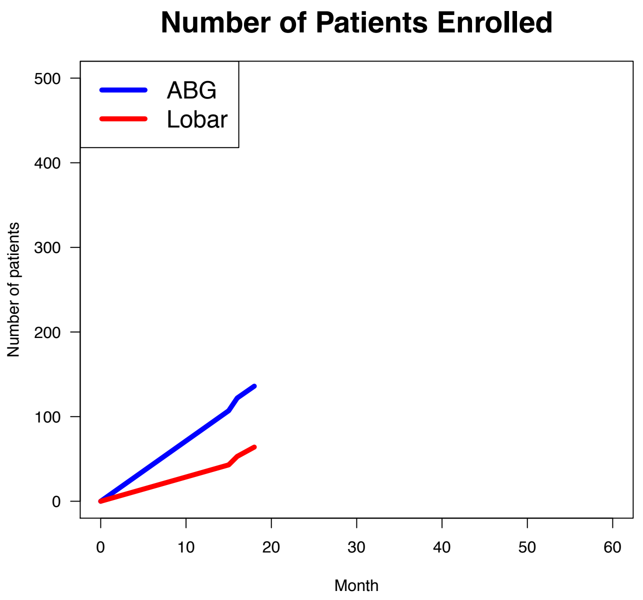

6.4.4 Example 4: Update 4

Table 31: Example 4: Update 4

Update	Month	Number Patients Enr	Compl	Treatment Benefit ABG	Lobar	All(SE)	Posterior Prob Fut(ABG)	Fut(Lb)	Succ(All)	Pval	Stopping Indicators Fut(ABG)	Fut(Lb)	Succ(All)
4	20	225	130	0.06	0.11	0.07 (0.06)	0.406	0.622	0.881	0.133	no	no	no

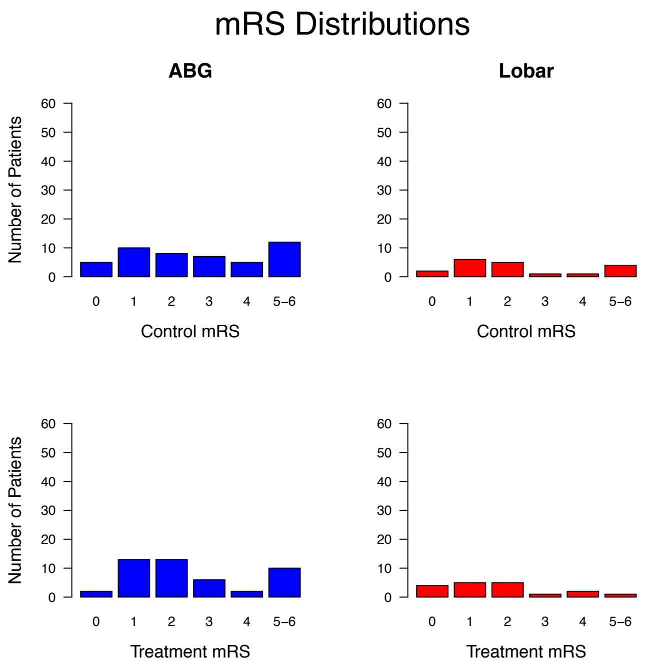

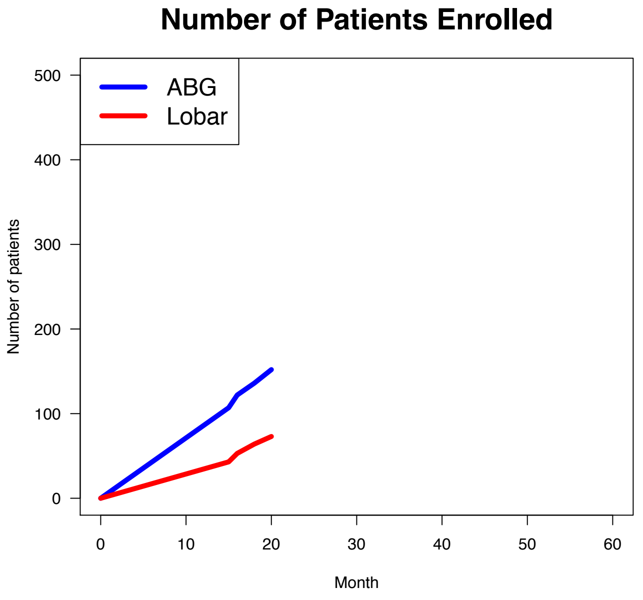

6.4.5 Example 4: Update 5

Table 32: Example 4: Update 5

Update	Month	Number Patients Enr	Compl	Treatment Benefit ABG	Lobar	All(SE)	Posterior Prob Fut(ABG)	Fut(Lb)	Succ(All)	Pval	Stopping Indicators Fut(ABG)	Fut(Lb)	Succ(All)
5	21	250	145	0.06	0.16	0.09 (0.06)	0.432	0.816	0.926	0.072	no	no	no

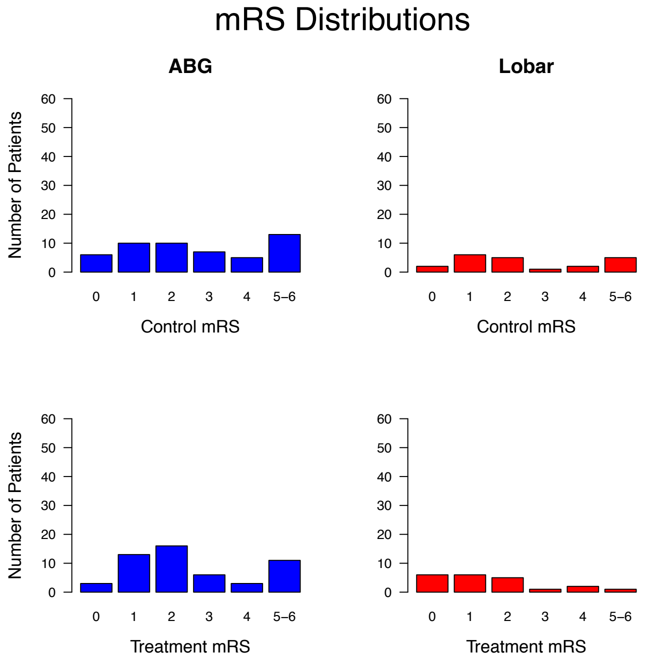

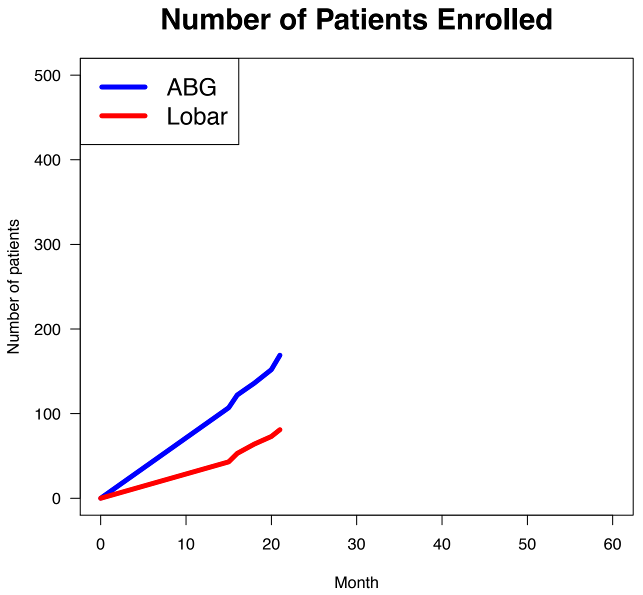

6.4.6 Example 4: Update 6

Table 33: Example 4: Update 6

Update	Month	Number Patients Enr	Compl	Treatment Benefit ABG	Lobar	All(SE)	Posterior Prob Fut(ABG)	Fut(Lb)	Succ(All)	Pval	Stopping Indicators Fut(ABG)	Fut(Lb)	Succ(All)
6	22	275	175	0.06	0.17	0.09 (0.06)	0.410	0.851	0.954	0.044	no	no	no

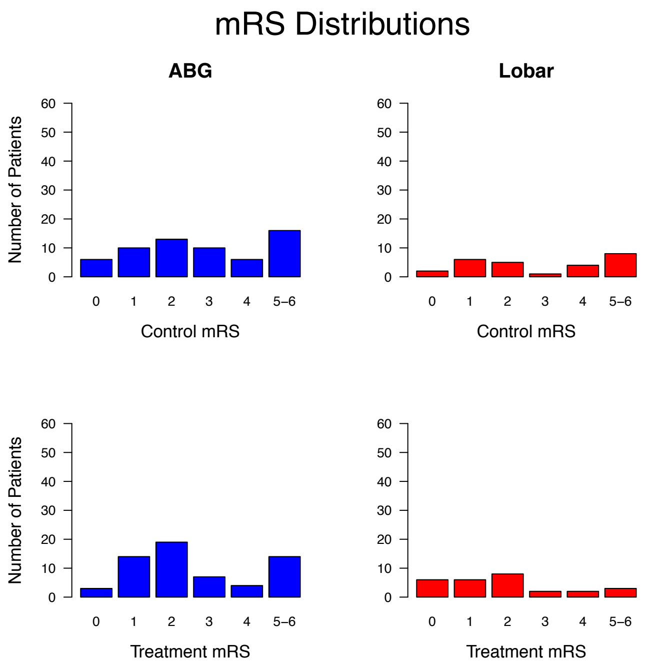

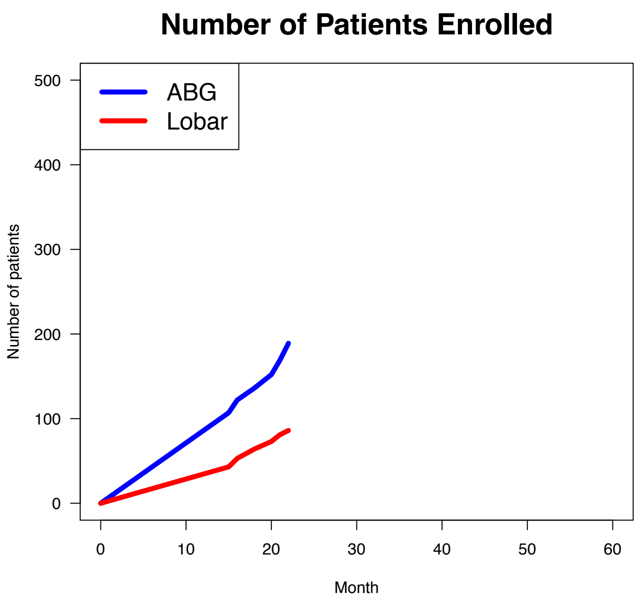

6.4.7 Example 4: Update 7

Table 34: Example 4: Update 7

Update	Month	Number Patients Enr	Compl	Treatment Benefit ABG	Lobar	All(SE)	Posterior Prob Fut(ABG)	Fut(Lb)	Succ(All)	Pval	Stopping Indicators Fut(ABG)	Fut(Lb)	Succ(All)
7	24	300	208	0.08	0.18	0.11 (0.05)	0.522	0.917	0.986	0.012	no	no	no

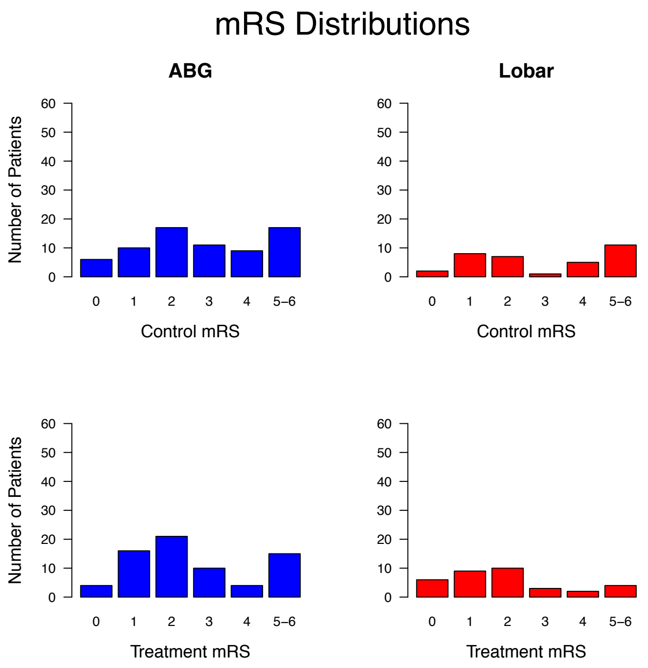

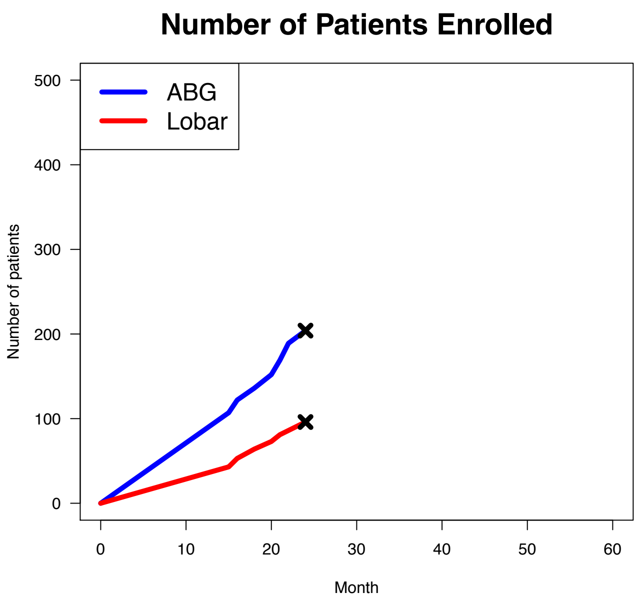

6.4.8 Example 4: Final

Table 35: Example 4: Final

Update	Month	Number Patients Enr	Compl	Treatment Benefit ABG	Lobar	All(SE)	Posterior Prob Fut(ABG)	Fut(Lb)	Succ(All)	Pval	Stopping Indicators Fut(ABG)	Fut(Lb)	Succ(All)
Final	30	300	300	0.09	0.17	0.11 (0.04)	0.634	0.931	0.996	0.003	no	no	yes

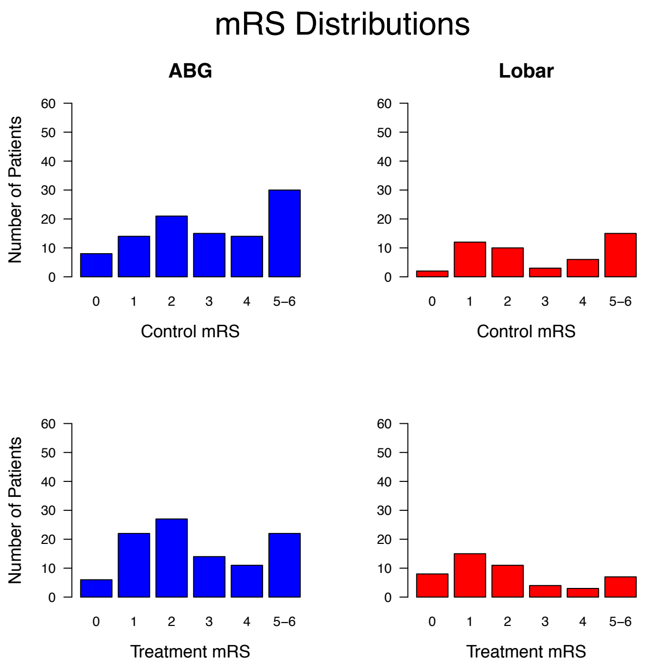

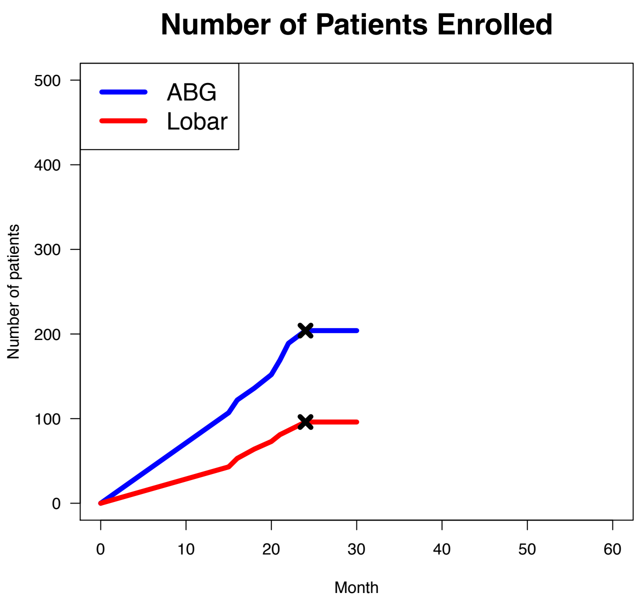

Supplement: Supplementary file 3 [file Data_Sheet_3.PDF]
